# Distinct lung cell signatures define the temporal evolution of diffuse alveolar damage in fatal COVID-19

**DOI:** 10.1016/j.ebiom.2023.104945

**Published:** 2023-12-23

**Authors:** Luke Milross, Bethany Hunter, David McDonald, George Merces, Amanda Thomson, Catharien M.U. Hilkens, John Wills, Paul Rees, Kasim Jiwa, Nigel Cooper, Joaquim Majo, Helen Ashwin, Christopher J.A. Duncan, Paul M. Kaye, Omer Ali Bayraktar, Andrew Filby, Andrew J. Fisher

**Affiliations:** a Newcastle University Translational and Clinical Research Institute, Newcastle upon Tyne, UK; b Newcastle University Biosciences Institute, Newcastle upon Tyne, UK; c Innovation Methodology and Application Research Theme, Biosciences Institute, Newcastle University, Newcastle upon Tyne, UK; d Department of Veterinary Medicine, University of Cambridge, Cambridge, UK; e Department of Biomedical Engineering, Swansea University, Wales, UK; f Imaging Platform, Broad Institute of MIT and Harvard, 415 Main Street, Boston, Cambridge, MA, USA; g Department of Cellular Pathology, Newcastle upon Tyne Hospitals NHS Foundation Trust, Newcastle upon Tyne, UK; h York Biomedical Research Institute, Hull York Medical School, University of York, York, UK; i Department of Infection and Tropical Medicine, Newcastle upon Tyne Hospitals NHS Foundation Trust, Newcastle upon Tyne, UK; j Wellcome Sanger Institute, Hinxton, Cambridge, UK; k Institute of Transplantation, Newcastle upon Tyne Hospitals NHS Foundation Trust, Newcastle upon Tyne, UK

**Keywords:** COVID-19, Post-mortem lung tissue, Diffuse alveolar damage, Immunopathology, Imaging mass cytometry

## Abstract

**Background:**

Lung damage in severe COVID-19 is highly heterogeneous however studies with dedicated spatial distinction of discrete temporal phases of diffuse alveolar damage (DAD) and alternate lung injury patterns are lacking. Existing studies have also not accounted for progressive airspace obliteration in cellularity estimates. We used an imaging mass cytometry (IMC) analysis with an airspace correction step to more accurately identify the cellular immune response that underpins the heterogeneity of severe COVID-19 lung disease.

**Methods:**

Lung tissue was obtained at post-mortem from severe COVID-19 deaths. Pathologist-selected regions of interest (ROIs) were chosen by light microscopy representing the patho-evolutionary spectrum of DAD and alternate disease phenotypes were selected for comparison. Architecturally normal SARS-CoV-2-positive lung tissue and tissue from SARS-CoV-2-negative donors served as controls. ROIs were stained for 40 cellular protein markers and ablated using IMC before segmented cells were classified. Cell populations corrected by ROI airspace and their spatial relationships were compared across lung injury patterns.

**Findings:**

Forty patients (32M:8F, age: 22–98), 345 ROIs and >900k single cells were analysed. DAD progression was marked by airspace obliteration and significant increases in mononuclear phagocytes (MnPs), T and B lymphocytes and significant decreases in alveolar epithelial and endothelial cells. Neutrophil populations proved stable overall although several interferon-responding subsets demonstrated expansion. Spatial analysis revealed immune cell interactions occur prior to microscopically appreciable tissue injury.

**Interpretation:**

The immunopathogenesis of severe DAD in COVID-19 lung disease is characterised by sustained increases in MnPs and lymphocytes with key interactions occurring even prior to lung injury is established.

**Funding:**

10.13039/100014013UK Research and Innovation/10.13039/501100000265Medical Research Council through the 10.13039/501100023449UK Coronavirus Immunology Consortium, Barbour Foundation, 10.13039/501100017475General Sir John Monash Foundation, 10.13039/501100000774Newcastle University, 10.13039/100010089JGW Patterson Foundation, 10.13039/100010269Wellcome Trust.

**Research in context**

**Evidence before this study**

• Lung damage in severe COVID-19 is highly heterogeneous yet studies examining spatial immune cell distinction in the evolution of discrete damage patterns are limited.
• Lung tissue is inherently collapsible which likely impacts cell count accuracy in previous spatial cytometric analyses.

**Added value of this study**

• This study used imaging mass cytometry with an airspace correction step and dedicated spatial immune cell distinction of separate damage patterns to investigate differences in immunopathology as diffuse alveolar damage progresses or the disease diverges.

**Implications of all the available evidence**

• The immunopathogenesis of severe DAD is characterised by mononuclear phagocyte, lymphocyte and neutrophil subpopulations.
• Important immune cell interactions can be identified by this technology prior to overt tissue damage.

## Introduction

Refractory respiratory failure is the leading cause of death in patients critically ill with COVID-19 ^1^ and immune-mediated acute lung injury rather than direct cytotoxic effects of SARS-CoV-2 infection appears central to disease severity.^2^ Characterisation of the host immune response at the lung tissue level using multimodal approaches is critical to understanding disease pathophysiology.^3^

Diffuse alveolar damage (DAD) is the predominant histological pattern in post-mortem lung tissue (PMLT) from cases of acute severe COVID-19^4^ and is considered a characteristic feature. DAD is routinely divided by pathologists into an acute exudative phase (EDAD), which may progress to a proliferative and organising phase (ODAD).^5^ DAD stages frequently coexist within a single patient^6^ as a temporally heterogeneous pathology. Alternate patterns of lung tissue damage are also recognised,^7^ including superimposed bacterial bronchopneumonia (BRON),^5^ pulmonary oedema consistent with acute cardiac failure (PO-ACF)^8^ and invasive pulmonary mycosis (IPM).^9^ How these different phases of DAD and the alternate patterns of lung injury influence the nature of the immune response is currently unclear.

Immune-mediated acute lung injury rather than the direct cytotoxic effects of SARS-CoV-2 infection itself appears to be central to severe or fatal COVID-19, evidenced by a topological dissociation between inflammatory and viral-positive areas,^2^ reduced or absent virus in late disease^10^^,^^11^ and that therapeutically, the inflammation-modulating glucocorticoid dexamethasone provides a significant mortality reduction in severe disease.^12^ The systemic immune response in COVID-19 shows major shifts in lymphoid and myeloid compartments as blood signatures of severe disease^13^ and the ‘competent’ immune profiles associated with mild COVID-19.^14^ However, these studies provide mere inferences to the cellular responses and architectural injury hidden at the tissue level where the end organ dysfunction occurs and detailed immunophenotyping of affected tissue is required to complete the picture.^15^, ^16^, ^17^

A suite of advanced pathology techniques were utilised early in the pandemic to dissect the shifts in immune and structural cells in COVID-19 post-mortem lung tissue (PMLT).^3^ Major emerging themes included a significant macrophage infiltration, expansion of T and B lymphocytes and mesenchymal and fibroblastic proliferation^15^^,^^18^ and intriguingly a topological dissociation between inflammatory and viral-positive areas.^2^ Published analyses of COVID-19 PMLT have significant limitations due to indiscriminate comparison of control tissue with an undifferentiated amalgam of COVID-19 ‘diseased tissue’.^3^ Furthermore, the metric commonly used to quantify immune cells in lung tissue, namely *cells per unit area of tissue section*, generally expressed as ‘cells/mm^2^’^15^^,^^18^, ^19^, ^20^ can be confounded by changes in airspace contributions to section area.

In this paper, we studied lung tissue from a cohort of 40 people who died with severe COVID-19 and analysed pathologist-guided lung tissue regions of interest (ROIs) representing the distinct temporal stages of DAD as well as alternate COVID-19-related disease patterns.

## Methods

### Tissue bank assembly and cohort description

**Fig. 1:**
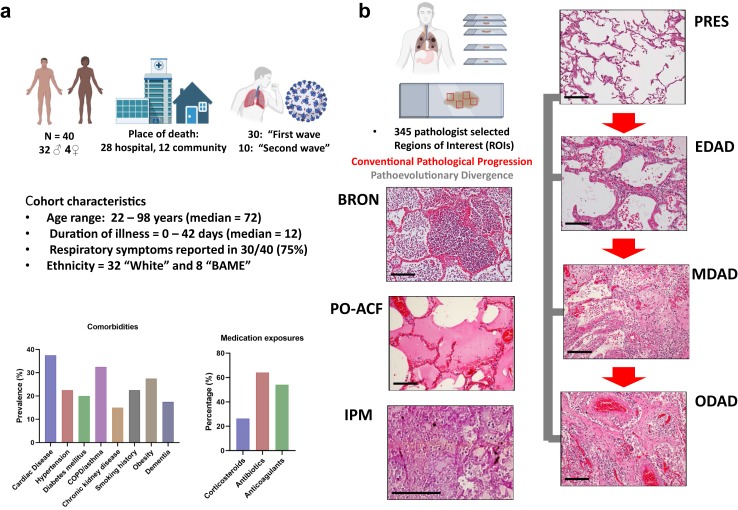
**Overview of the cohort demographics and histological model.** a) A graphical summary of the cohort composition and key clinical metadata, including comorbidities and exposure to medication. b) A graphical and histological summary of the different pathology states present in the cohort as identified by expert pathologist input (H&Es at ×100 magnification except for IPM which is at ×200 magnification). Note that the conventional progression stages are shown with red directional arrows and that the pathoevolutionary divergent stages are linked by grey lines to denote that progression and origin are unknown with respect to conventional stages of pathology. Histology images are derived from H&E stained FFPE serial tissue sections adjacent to those used for IF and IMC analysis. Please also note that “PRES” pathology falls in to two classes; derived from SARS-CoV2 infected and uninfected tissue. Scale bar = 100 μm. Some figure components were created with Biorender.com.

Lung tissue was obtained via autopsy by the Letulle method from three United Kingdom Tissue Biobanks from individuals with clinical and microbiological evidence that COVID-19 disease was the primary precipitant of death with a period of recruitment from of 1 year from April 2020 to April 2021. Data collection and analysis extended from April 2021 until December 2022. Sample size was determined by tissue donor availability from these biobanks, with all cases available being included. All patients were confirmed SARS-CoV-2-positive by reverse transcription polymerase chain reaction (RT-PCR) of nasopharyngeal and/or direct lung tissue swabs at autopsy. We included a cohort of 40 adults, whose clinical metadata was obtained from electronic medical records and post-mortem reports (Fig. 1, Table 1, Tables S1 and S2). Metadata included basic subject demographics, prevalence of comorbidities, clinical course and exposure to medications which was defined as any treatment with a corticosteroid, antibiotic or therapeutic anticoagulant prior to death. Architecturally preserved control tissue, henceforth referred to as ‘PRESneg’ was obtained from unused lung donors which did not proceed to transplantation (n = 2). Two cases of disease-free control lung tissue were obtained from donor lungs not used in transplantation where the donor's next of kin consented for use for NHS REC approved research.

**Table 1:** Characteristics of the cohort.

Cohort Characteristics—n = 40	
**Age (years)**	
Range	22–98
Interquartile range	61.5–79.75
Median	72
**Sex**	
Female	8 (20%)
Male	32 (80%)
**Ethnicity**	
BAME	8 (20%)
White	32 (80%)
**Place of death**	
Community	12 (30%)
Hospital	28 (70%)
**Pandemic wave**	
First	30 (75%)
Second	10 (25%)
**Pre-existing comorbidities**	
Cardiac disease	15 (37.5%)
Hypertension	9 (22.5%)
Diabetes mellitus	8 (20%)
COPD/Asthma	13 (32.5%)
CKD	6 (15%)
Smoking history	9 (27.5%)
Obesity	11 (27.5%)
Dementia	7 (17.5%)

### Tissue section preparation and ROI selection

Formalin-fixed paraffin-embedded (FFPE) lung tissue blocks from multiple lung regions were serially cut and mounted onto slides. Formalin fixation was for 72 h as per local protocols given the biohazard risk associated with lung tissue from patients with COVID-19. Multiple lung blocks were taken from each patient to provide ample tissue for analysis, with number taken at the discretion of pathologists from each centre, ranging from 2 to 11 per patient (median = 7). Lobe selection for block curation was also at the pathologist's discretion however multiple pulmonary lobes were represented in all cases. The H&E-stained primary slide from each serial deck was scanned onto the open-source digital online pathology platform OMERO (‘The Open Microscopy Environment’). ROIs were selected under guidance of a consultant histopathologist with cardiothoracic expertise with sizes ranging from 0.25 mm^2^ (500 μm × 500 μm) to 1 mm^2^ (1000 μm × 1000 μm). ROI classifications included the temporal phases of DAD, bronchopneumonia (‘BRON’), pulmonary oedema consistent with acute cardiac failure (‘PO-ACF’) and invasive pulmonary mycosis (IPM). ROIs were also selected from DAD-free, ‘preserved’ regions of lung tissue from these individuals who were SARS-CoV-2 infected (titled PRESpos), as well as from donors who were SARS-CoV-2 negative (PRESneg).

DAD was divided based on published histological criteria^21^ into exudative DAD (EDAD), organising DAD (ODAD) and mixed (or ‘intermediate’) diffuse alveolar damage (‘MDAD’). Of these criteria, selection was weighted by a primary ‘hallmark’ characteristic in the context of secondary supportive features (Table S3).

### Manufacture of control tissue MicroArray (TMA) material

To provide positive and negative staining controls for all 40 antibodies in our panel as well as provide empirical controls for batch effects we prepared FFPE Tissue MicroArrays (TMAs) blocks, composed of human tonsil tissue, as well as both SARS-CoV2-infected (BetaCoV/England/2/2020, obtained from the UK Health Security Agency)) and uninfected Vero E6 cells. SARS-CoV-2 is a Hazard Group 3 pathogen (Advisory Committee on Dangerous Pathogens, UK), as such infections were performed in a dedicated Containment Level 3 (CL3) facility by trained personnel as described in Hatton et al. (2021).^22^ Vero E6 cells were commercially-obtained (ATCC Cat# CRL-1586, RRID:CVCL_0574) with short tandem repeat testing confirming authenticity and purity as well as Mycoplasma testing performed by the vendor. Vero E6 cells were seeded in a 175 mL flask until 90% confluent then infected with 1.5 × 10ˆ6 PFU/mL of SARS-CoV-2 diluted in 2% FCS DMEM. The inoculum was removed after 2 h and replaced with 30 mL of warm 2% FCS DMEM. After 72 h, supernatant was collected and centrifuged at 2000 rpm for 30 min at 4′C. Supernatant was removed and the pellet was then resuspended in 4% formaldehyde for 1 h at RT. Cells were then spun at 500g for 5 min and resuspended in 70% alcohol solution/IMS. Cell pellets were processed and paraffin embedded at the Novopath Research Service (NovoPath, Department of Pathology, Newcastle Hospitals NHS Foundation Trust, Newcastle upon Tyne, UK). 2 mm cores from paraffin embedded SARS-CoV-2 infected Vero E6 cells were embedded alongside 2 mm cores of uninfected Vero E6 cells and Tonsil tissue, to produce a control tissue microarray block that was mounted on super frost slides and processed/stained alongside each batch of patient samples.

### Antibody panel design, conjugation and antigen retrieval for imaging mass cytometry analysis

The 40-plex antibody panel identifying the immune, signalling and stromal components in the surrounding microenvironment of COVID-19 post-mortem lung tissue is described in Table S4. Antibodies were commercially available with the exception of anti-B7 and anti-C3-30 obtained and previously validated by academic collaborators.^23^ All antibodies used in this study were pre-validated for performance using Tris EDTA pH9 “Heat-Induced Epitope Retrieval” (HIER) two colour immuno-fluorescence (IF) and conjugated (where necessary) to lanthanide metals and fully validated. Metal tags were paired with antibodies based on the relative staining intensity of each marker as determined by IF following the rules of “best practice” for CyTOF panel design^24^ using the Maxpar X8 metal conjugation kit following manufacturer's protocol (Standard Biotools, CAT#201300). Antibodies conjugated to platinum isotopes 194 Pt and 198 Pt were conjugated as described in Mei et al., 2015.^25^ Conjugation success was determined by measuring antibody recovery post-conjugation, metal addition by binding the antibody to iridium labelled antibody capture beads AbC™ Total Antibody Compensation Beads (Thermo Fisher, USA, CAT#A10513) and acquiring on a Helios system (Standard Bio-tools, USA), and finally a retained ability to recognise antigen post-conjugation using either a two layer IF or directly by IMC using the Hyperion imaging module (Standard Bio-Tool) connected to the Helios.

### Hyperion (IMC) set up, quality control (QC) and sample acquisition

Prior to each slide acquisition, the Hyperion Tissue Imager was calibrated/QC'd to achieve reproducible sensitivity based on the detection of 175 Lutetium by ablating a single multi-element-coated “tuning slide” (Standard Biotools, USA) using the manufacturer's “auto tune” application. After tuning, TMA control and experimental slides were loaded onto the Hyperion system to create Epi-fluorescence panorama images of the tissue surface and regions of interest (ROI) were set based on OMERO annotations. A small region of tonsil tissue was targeted to test that the chosen laser power was able to ablate the entire tissue depth. First, three 0.25 mm^2^ ROIs, one per TMA control, were ablated followed by ROIs from the post-mortem cases with ROI sizes ranging from 0.25 to 1 mm^2^. Ablations were performed at 200Hz laser frequency to create a resultant MCD file containing all data from all ROIs for a given slide/case. MCD files were then opened in MCD Viewer software (Standard Bio-Tools) to perform a qualitative, visual QC of the staining intensity and pattern with the initial IF images as a benchmark. All images were exported as 16-bit single multi-level TIFFs using the “export” function from the “file” menu. For efficiency, all open collection channels from the experimental acquisition template (in this case, 60, including several “Blank” channels for QC purposes) from all ROIs were left selected and any image/channel removal was dealt with later in the analysis. The multi-level 16-bit TIFF images were then input into our OPTIMAL pipeline^26^ for data exploration at the single cell, spatial level.

### Cell segmentation, feature extraction, parameter correction/normalisation and FCS file creation

Cell segmentation was performed as per the OPTIMAL pipeline^26^ using Ilastik. Output nuclear probability maps were input into CellProfiler to segment cell nuclei, that in turn acted as seeds for cell segmentation using a propagation algorithm based on the EPCAM signal (Figure S1). Single cell objects were measured for mean intensity in each of the labelled channels and corrected for metal signal “spillover” according to a previously described approach.^27^ An *arcsinh* transformation cofactor (c.f.) of 1 was applied to all metal signal parameters. Batch effect correction was performed using Z-score normalisation on the *arcsinh* c.f. 1 transformed data (Figure S2). We also added additional metadata to the files such as batch number, to be accessible and plot–able parameters for subsequent analysis. Final matrix data was converted to FCS file format within the MATLAB pipeline.

### Visualisation, clustering and spatial exploration of single cell IMC data

FCS Express software (Version 7.14.0020 or later, Denovo software by Dotmatics, USA) was used as outlined in the OPTIMAL method.^26^ Briefly, the FCS files created from the segmentation pipeline of each ROI were loaded as a single merged file. Gates were created on batch, biobank source, pathology class etc. to aid with meta-analysis. SARS-CoV-2 spike and nucleocapsid protein expression was determined for each of the 8 pathology classes on a per cell basis using histogram displays, and the population means were compared to the SARS-CoV-2 infected and mock infected Vero E6 cell TMA controls. Single cell data structure for all 38 positive signals (*arcsinh* c.f. 1 transformed and Z score normalised) was displayed by creating a PacMap dimensionality reduction plot as described previously.^26^ To identify resident cell types and states the same 38 transformed and normalised metal parameters were used as input in to the FLOWSOM clustering algorithm as outlined previously.^26^^,^^28^ The default 100 SOMs (clusters) were merged using a hierarchical approach to 40 consensus SOMs (cSOMS). The 40 cSOMs were further merged to 25 final “tier 2” clusters based on expert annotation and *a priori* knowledge from heat map interrogation. “Tier 2” clusters were then merged to 10 “high level” cell types denoted as “tier 1”. Spatial neighbourhood analysis for tier 1 and tier 2 clusters was performed as outlined in Hunter et al. (disc outgrowth of 5 pixels and 100 iterations of randomly mapping cells back on to the segmentation maps).^26^ This was dependent on the scale of the number of nearest neighbours considered. Statistically significant interactions between cell types were determined by comparing spatial cell iterations and those obtained by the random permutations of the cell positions. If differences were detected in the original data compared to a 90% confidence interval of the random iterations, then a significant difference (interaction or avoidance) was listed for that cell type. Statistically significant interactions between cell types were determined by comparing each cell type's nearest neighbour phenotypes to that expected if the cells in that field of view were randomly positioned. This is carried out using a Monte Carlo simulation which takes the identical number of cell phenotypes and positions them randomly at cell positions in the field of view, each cell's nearest neighbour phenotypes are then calculated. We carried out 100 iterations of this procedure and a significant difference (interaction or avoidance) is detected in the original data compared to a 90% confidence interval of the random iterations for each field of view. The heatmap shows the fraction of the fields of view where that interaction was determined to be significant. A 90% confidence interval allows more permissive measurement of possible interactions of lower frequency cell types and accounts for the variability across ROIs. This analysis workflow is based on a tested approach^26^ optimised on tonsillar tissue with known structure and cellular interactions. These positive, neutral, and negative interactions were then averaged to create the overall heatmap for the condition (i.e., pathology, region, etc.). These interactions were assessed across all 8 pathology classes.

### Airspace correction and normalisation of cell counts by tissue area

The metric commonly used to quantify immune cells in lung tissue, namely *cells per unit area of tissue section*, generally expressed as ‘cells/mm^2^’^15^^,^^18^, ^19^, ^20^ can be confounded by changes in airspace contributions to section area. To compensate for the confounding effect of airspace obliteration, we developed a correction method to standardise cellularity when describing distinct lung cell signatures in COVID-19 PMLT. For each ROI, the percentage of airspace was determined using the following equation (1):

1 $$\left(1-\left(\left(\frac{\sum ca}{10^{6}}\right)/ROI\right)\right)\times 100$$

where *ca* = the area of each single cell in each ROI in μm^2^ and *ROI* = the area of the imaged ROI in mm^2^. Furthermore, the area of cellular tissue in mm^2^ (first part of Equation 1 above) was used to normalise all cell counts to account for any artificial increases due to tissue collapsing into the imaged ROI due to loss of air gaps (henceforth referred to as *airspace correction*).

### Statistics

GraphPad Prism version 9.5.0 was used for all statistical analyses. For differential analysis of cell counts and percentages at tier 1 and tier 2, data distribution was first assessed by the Shapiro–Wilk normality test. The non-parametric Kruskal–Wallis one way analysis of variance test was next performed. Dunn's tests were used for post-hoc pairwise comparisons with each P value adjusted to account for multiple comparisons with a family-wise alpha threshold and confidence level of 5. Results were therefore considered statistically significant where P < 0.05. Graphical data generated by GraphPad Prism 9.5.0 was represented in the format of median, interquartile range (IQR) and range.

### Ethics

Human samples used in this research project were partly obtained from the Newcastle Hospitals CEPA Biobank and their use in research is covered by Newcastle Hospitals CEPA Biobank ethics—REC 17/NE/0070. Control samples used in this research project were also obtained from this source and covered for use by REC 16/NE/0230. Additional human samples used in this research project were obtained from the Imperial College Healthcare Tissue Bank (ICHTB). ICHTB is supported by the National Institute for Health Research (NIHR) Biomedical Research Centre based at Imperial College Healthcare NHS Trust and Imperial College London. ICHTB is approved by Wales REC3 to release human material for research (22/WA/0214). Additional human samples used in this research project were obtained from the ICECAP tissue bank of the University of Edinburgh. ICECAP is approved by the East of Scotland Research Ethics Service to release human material for research (16/ED/0084). Work on these samples at the University of York was approved by the Hull York Medical School Ethics Committee (20/52). Human samples from all centres were obtained with written next of kin consent.

### Role of funders

Funders did not have any role in the study design, data collection, data analyses, interpretation or writing of reports.

## Results

### Demographics and clinical features

Lung tissue was analysed from 40 individuals who died with severe COVID-19 (8F/32M). Median age at death was 72 years (IQR = 61.5–79.75 years), with 28/40 (70%) dying in hospital and 12/40 (30%) dying in the community. Median duration of illness was 12 days (IQR = 8.75–22.25 days; duration of illness data known in 35/40 cases). 30/40 (75%) died in the ‘first wave’ of the pandemic (before 1st October 2020) and 10/40 (25%) died in the ‘second wave’ defined by increasing predominance of the alpha (B.1.1.7) variant.^29^ None were vaccinated against SARS-CoV-2. Autopsy was performed between same-day and seven days (median = 3 days) after death. All were SARS-CoV-2 positive by RT-PCR on pharyngeal or direct lung sampling. The cause of death in all cases was deemed primary respiratory failure with severe COVID-19 as the major antecedent cause (with or without superimposed bacterial or fungal infection) in all but two cases where heart failure with severe COVID-19 as the major antecedent cause occurred. Table 1, Table S1 and Figure S1 show cohort demographics, comorbidities and disease characteristics. Spike and nucleocapsid proteins have previously been detectable in COVID-19 PMLT analysed by IMC.^15^ However, using single cell expression data in positive and negative infected cultured cells as controls, we did not detect spike or nucleocapsid protein in any of the pathology phenotypes (Figure S3). This could be related to insufficient sensitivity of IMC analysis or support a ‘hit and run’ hypothesis whereby immunopathology is topically dissociated from virus.^2^

### Histopathological assessment reveals pathological heterogeneity

Regions of interest (ROIs) were selected representing the temporal phases of DAD and alternate pathological patterns as shown in Fig. 1. DAD was the commonest histopathological phenotype, identified in 29/40 cases (73%). Of these, 17 patients (59%) showed DAD in different evolutionary phases, indicating significant intra-patient temporal heterogeneity. 7 cases were predominantly BRON, 3 cases PO-ACF and one case IPM. The three cases which were predominantly PO-ACF were deemed to have died following heart failure with severe COVID-19 as the major antecedent cause. A total of 345 ROIs were selected for analysis. The number of ROIs selected in each pathological pattern is found in Table S2. There were no patients who were SARS-CoV-2 positive who did not have any lung tissue pathology.

### Single cell analysis reveals that airspace obliteration not increased cellularity, defines DAD progression

**Fig. 2:**
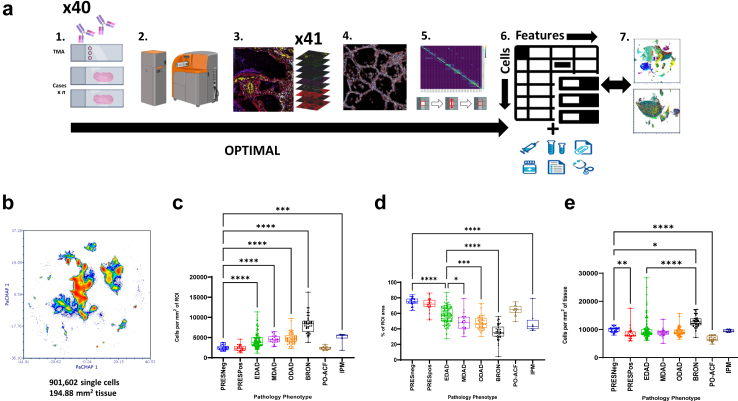
**OPTIMAL analysis of single cells in COVID19 PM lung tissue reveals a progressive loss of lung air space leading to elevated cellularity due to tissue obliteration rather than a *de* novo cellular influx.** a) PM lung tissue slides were stained with a panel of 40 metal tagged antibodies alongside an additional control TMA slide (1). The pathologist-marked ROIs were then set using an OMERO reference image and ablated using a Hyperion IMC system (2) to produce a set of 41 multispectral images (3) that were segmented to single cell data (4), corrected for spill-over and other factors that could affect clarity (5) and converted to FCS file format with additional key metadata added (6). Batch effect was determined and corrected for using a z-score normalisation approach (7). b) A PacMap dimensionality reduction plot for all 901,602 single cells representing ∼195 mm^2^ of COVID19 PM lung tissue. c) Cell counts per mm^2^ of the ablated ROI area for each of the 8 pathology classes. d) A graph showing the % of air space within each ROI as a function of pathology class. e) A graph of the cell counts per mm^2^ of actual lung tissue in each ROI. Differences between pathology classes were considered statistically significant where P ≤ 0.05 (Kruskal–Wallis test). ∗P ≤ 0.05, ∗∗P ≤ 0.01, ∗∗∗P ≤ 0.001, ∗∗∗∗P ≤ 0.0001. The whiskers of all box-and-whisker plots represent the range of data for all similar graphs in this Figure and ongoing.

345 ROIs, covering ∼195 mm^2^ lung tissue area, were ablated by imaging mass cytometry and single cell analysis was performed using the OPTIMAL approach^26^ (Fig. 2a, Figure S1). The pipeline included normalisation for batch effect from both run and tissue source (Figure S2). This process generated a total output of ∼901k single cells (Fig. 2b).

Statistical analyses of cellularity comparing distinct pathology types was performed using total cells normalised to area of each ROI (Kruskal–Wallis test, significant where P < 0.05) (Fig. 2c). There were significant increases in cellularity as the temporal phases of DAD progressed from preserved tissue, and BRON had the highest. However, it was unclear whether increased cellularity in DAD was related to actual cellular influx, or airspace obliteration leading to an increase of tissue within the ROI, or a combination of both. For example, Fig. 2d demonstrates striking loss of airspace across DAD progression. To account for this, we normalised cell counts by actual cellular tissue area by airspace correction, as opposed to ROI area. Fig. 2e demonstrates the effect of airspace correction in nullifying the increased cellularity previously seen across DAD, indicating that this was confounded by airspace obliteration. Notably, time to post-mortem had no influence on overall cell density when comparing ROIs from ‘early’ cases (≤3 days, n = 24) to ‘late’ cases (>3 days, n = 16) (data not shown).

### Increases in mononuclear phagocytes and lymphocytes and not neutrophils define the immune signature of COVID-19 DAD progression

**Fig. 3:**
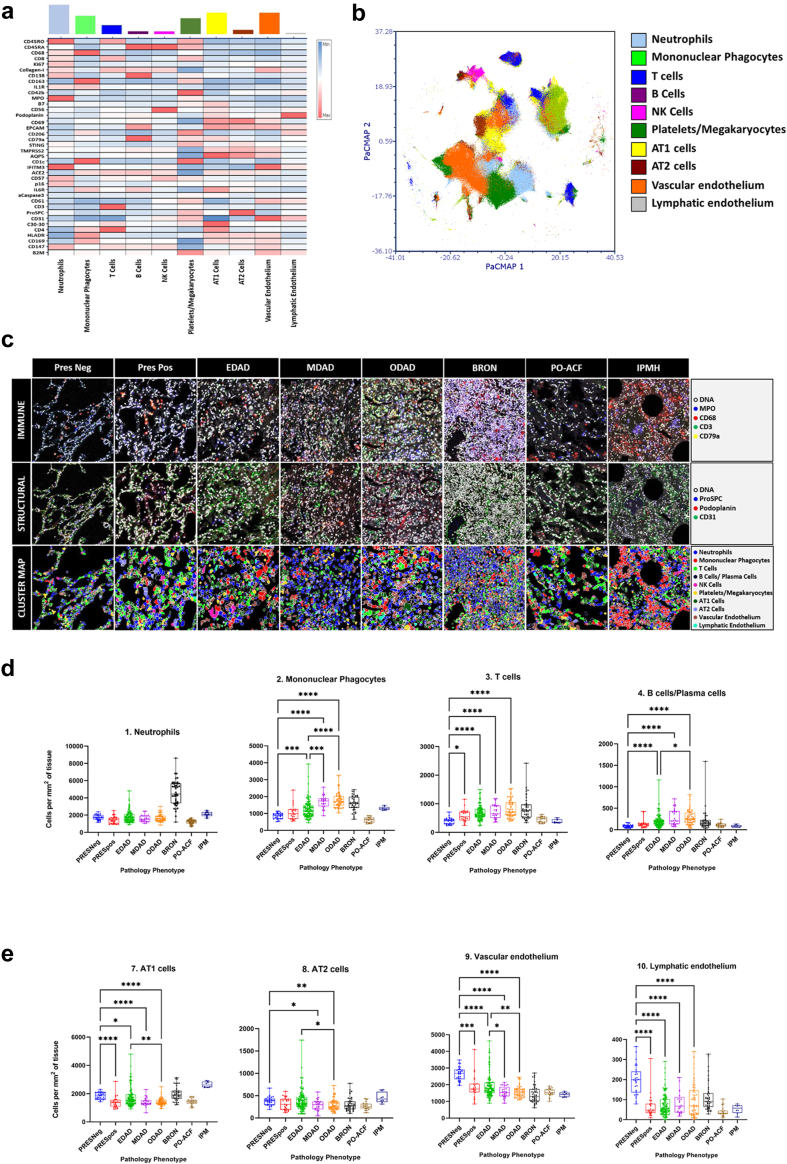
**Analysis of Tier 1 cell type clusters reveals key immune and structural cell signatures defining temporal stages of DAD and alternate pathology classes.** a) A heat map showing the median Z-score normalised values for all 38 phenotypic and functional markers (SARS-CoV2 Spike and Capsid were deemed to be negative so not used) for tier 1 level clusters. The coloured bars denote the frequency of each cluster across the entire single cell data set for all 8 pathology classes. b) A PacMap of the single cell level data coloured by tier 1 cluster as shown in the associated legend. c) Upper panels show pseudo-coloured overlaid key immune cell markers for representative ROIs from all 8 pathology classes. Middle panels show pseudo-coloured overlaid key structural cell markers for the same 8 representative ROIs. Lower panels show cluster maps for the same 8 representative ROIs with all 10 tier 1 clusters. Each column corresponds to duplicate ROIs. d) Graphs showing the cell counts per mm^2^ of lung tissue per ROI for key tier 1 immune cell types. From left to right; neutrophils, mononuclear phagocytes, T cells and B/Plasma Cells. e) Analogous graphs as shown in D but for major tier 1 structural cell types. From left to right; AT1, AT2, vascular endothelium and lymphatic endothelium. Differences between pathology classes were considered statistically significant where P ≤ 0.05 (Kruskal–Wallis test). ∗P ≤ 0.05, ∗∗P ≤ 0.01, ∗∗∗P ≤ 0.001, ∗∗∗∗P ≤ 0.0001.

Our high-level (Tier 1) analysis of immune and structural cells generated 10 consensus clusters (Fig. 3a, Figure S4), which were substantially discrete when mapped back to a PacMap dimensionality reduction plot (Fig. 3b).

We analysed pathology phenotypes for their Tier 1 immune cell airspace-corrected cellularity (Fig. 3d and e) and proportions (Figure S5a) (Kruskal–Wallis test, significant where P < 0.05). Neutrophils, alongside a modest rise in mononuclear phagocytes, were seen in BRON but not DAD.

Mononuclear phagocyte and lymphocyte infiltration represent the predominant immune cell hallmarks of COVID-19 DAD, with both showing increased proportions and airspace-adjusted cellularity as DAD evolved (Fig. 3c and d). Lymphocyte increases involved both CD4+ and CD8+ T cells and B-cells/plasma cells. Analysis of neutrophils with cells/mm^2^ tissue *prior to* airspace correction was misleading, as this metric suggested an increase in DAD classes compared to PRESneg (Figure S6). However, when applying proportion and airspace-adjusted cellularity metrics, no clinically meaningful differences in neutrophils were seen across any of the temporal phases of DAD and when compared with preserved tissue.

Subgroup analysis was next performed with a focus on EDAD ROIs, with data points coded by sex, ethnicity, pandemic wave and age (<70 vs ≥70 years old). When our PacMap dimensionality reduction plot was coloured for these demographic differences, no clear difference in data spread was seen (Figure S7). Comparisons between airspace-corrected cellularity were made for neutrophils, MnPs, T cells and B cells/plasma cells coded for sex, ethnicity, pandemic wave and age demonstrated only subtle differences including increased neutrophils and T cells in second wave compared to first wave EDAD ROIs which might be biologically meaningful if there was a difference in strain between waves and increased T cell infiltration in male compared to female EDAD ROIs which is less likely to be biologically meaningful. No differences were seen between the <70 and ≥70-year-old age cut-offs).

### Progressive loss of alveolar epithelial cells (AECs), vascular endothelial and lymphatic endothelial cells is seen in progressive COVID-19 DAD

A reduction in numbers of AT1 cells from PRESneg to EDAD and from EDAD to ODAD was identified (Fig. 3e). AT2 cells maintained stable proportions with respect to preserved tissue however there was a meaningful decrease in MDAD and ODAD compared to PRESneg. Vascular endothelial cells and lymphatic endothelium also decreased as DAD progressed. Fig. 3c shows raw IMC images and cluster maps for Tier 1 populations in all pathology classes, visually displaying variations in immune and structural populations.

### Individual immune cell phenotypes characterise temporal phases of the COVID-19 DAD continuum

**Fig. 4:**
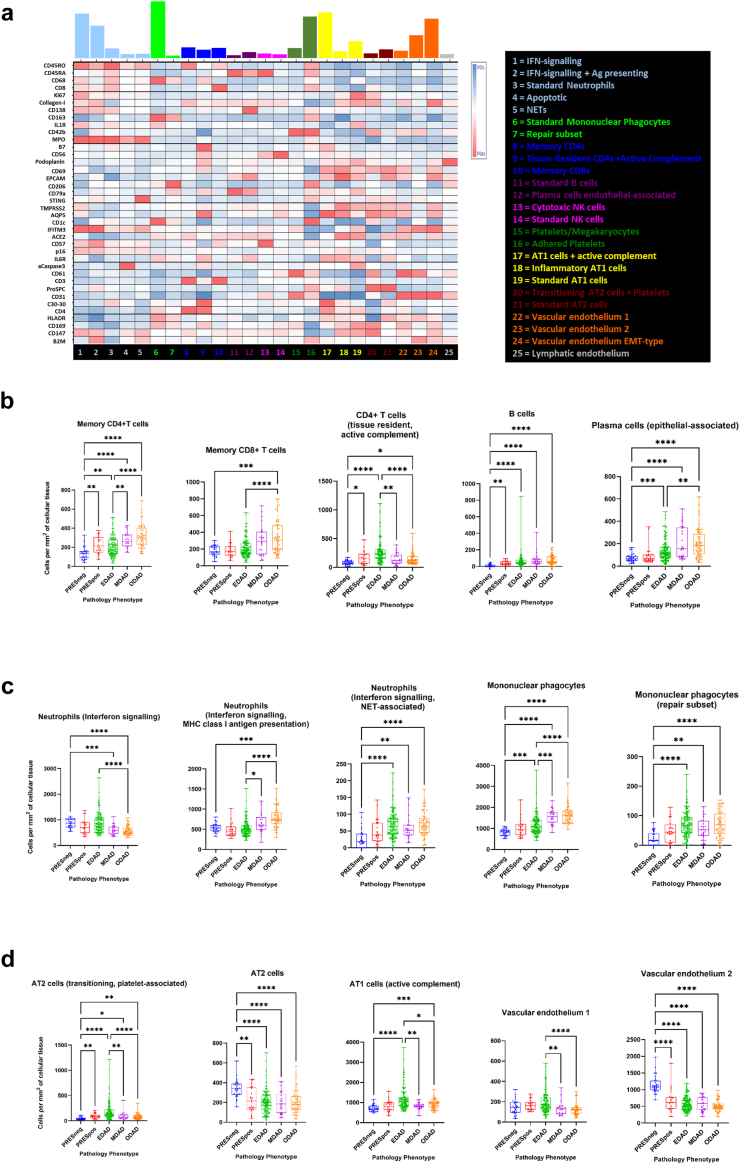
**Tier 2 cluster analysis reveals a unique set of cell signatures linked to DAD progression.** a) A heat map showing the median Z-score normalised values for all 38 phenotypic and functional markers (SARS-CoV2 Spike and Capsid were deemed to be negative so not used) for tier 2 level clusters. The coloured bars denote the frequency of each cluster across the entire single cell data set for all 8 pathology classes. The cluster ID is given by the number below the column (cluster) and denoted in the legend. b) Graphs showing the cell counts per mm^2^ of lung tissue per ROI for key tier 2 lymphocytic cell types. From left to right; memory CD4 T cells, memory CD8 T cells, CD4 T cells with active complement and tissue resident features, B cells and epithelial-associated plasma cells. c) Graphs showing the cell counts per mm^2^ of lung tissue per ROI for key tier 2 non-lymphocytic immune cell clusters. From left to right; neutrophils with signatures of interferon signalling and MHC-class 1 presentation, neutrophils with interferon signalling and NET-associated signatures, mononuclear phagocytes and mononuclear phagocytes with a repair-promoting signature. d) As in B and C but showing key tier 2 structural cell clusters. From left to right; AT2 cells with a transitioning and platelet associated signature, AT 2 cells, AT1 cells with active complement, vascular endothelium 1 (CD61+ CD31+ cells mapping to capillaries) and vascular endothelium 2 (CD61+ CD31+ cells mapping to vascular structure outlines of greater size than capillaries). Differences between pathology classes were considered statistically significant where P ≤ 0.05 (Kruskal–Wallis test). ∗P ≤ 0.05, ∗∗P ≤ 0.01, ∗∗∗P ≤ 0.001, ∗∗∗∗P ≤ 0.0001.

We next used a 38-marker panel with the FLOWSOM algorithm to identify 25 “Tier 2” clusters and used a Z score normalised heat map to annotate the cell types and states (see Fig. 4a). We then focused on the PRES and DAD groups and the airspace-corrected cellularity metric, making comparisons between pathology groups (Kruskal–Wallis test, significant where P < 0.05). Figures S8–S10 contain analyses by other metrics.

With respect to adaptive immune cells (Fig. 4b), the rises in lymphocytes seen in Tier 1 analysis were accounted for by increased memory CD4+ T cells, memory CD8+ T cells, CD4+ T cells, B cells and plasma cells. Plasma cell infiltration was particularly marked as DAD progressed which is highly biologically relevant. With respect to innate immune cells, although total neutrophil infiltration is not a hallmark of COVID-19, two phenotypic subsets of neutrophils characterised by interferon signalling (IFITM3^HI^ and STING^HI^), MHC class I antigen presentation (beta-2 microglobulin^HI^) and neutrophil-extracellular traps were markedly increased as DAD progressed as shown in Fig. 4c. An inflammatory subset of mononuclear phagocytes (IL1R^HI^, IL6R^HI^ and HLA-DR^HI^) also increased in number from PRESneg to EDAD and again from EDAD to ODAD. A second cluster of macrophages, phenotypically consistent with M2 polarisation (CD206^HI^) were increased from PRESneg to DAD phases. No further increases were noted between EDAD, MDAD and ODAD, perhaps suggesting an exhausted reparative process.

Amongst the structural clusters (Fig. 4d), we observed an AT1 and a CD4+ T cell cluster with markers suggestive of activated complement activity (C3–30^HI^ and B7^HI^) that were elevated in EDAD compared to PRESneg and ODAD. SARS-CoV-2 can activate the complement system via the classical, lectin and alternative pathways or indirectly through endothelial injury and thromboinflammation^30^ and our results suggest a biologically plausible association with AT1 cells and a CD4+ T cells especially during early DAD. A subset of AT2 cells is seen falling as disease progresses which may indicate a known transition process to an AT1 phenotype to replace those lost in the tissue.^31^

### Critical immune cell interactions are established early, prior to overt tissue damage

**Fig. 5:**
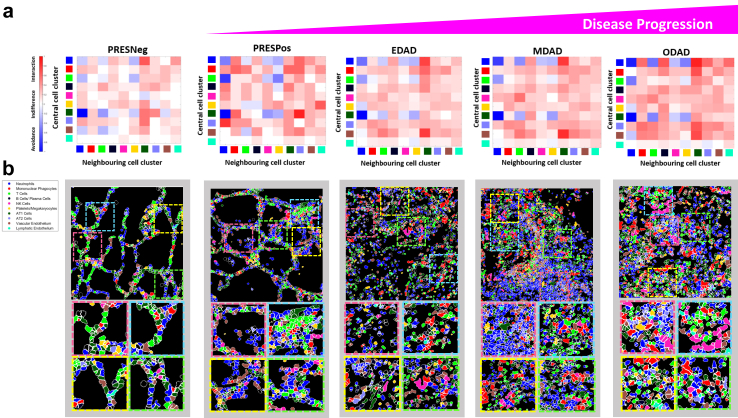
**Spatial neighbourhood analysis of tier 1 clusters reveals key interactions and avoidances that correlate with DAD initiation and progression.** a) Heat maps for each of the 5 major pathology classes involved in classical disease progression showing the significance of interaction, avoidance or indifference for all 10 tier 1 clusters as per the legend. b) Representative cluster maps with coloured boxes denoting areas of focus demonstrating highly biologically relevant interactions. Magnified duplications of coloured boxes are provided in the lower part of the panel. A section of the panel component representing EDAD in Fig. 5 is a duplicate of the panel component representing the EDAD cluster map in Fig. 3.

Analysis of cellular neighbourhoods at Tier 1 immune cell level is showed in interaction/avoidance heat maps for PRES and DADs (Fig. 5). All other pathologies are shown in Figure S11 for Tier 1 and in Figure S12 for Tier 2. Quantitative comparison of a select group of highly biologically relevant interactions highlighted in Fig. 5 are shown in Figure S13. Marked differences occurred between PRESneg and PRESpos, suggesting critical interactions are established early, prior to overt tissue damage. Notably, in PRESpos, neutrophils appear to interact more with AT2 cells, the primarily infected cell in lung tissue in previous literature,^32^ and less with AT1 cells. The most striking specific difference was an increased interaction between neutrophils with interferon signalling and MHC class I antigen presentation markers and AT2 cells (Figure S12). Additionally, we noted mononuclear phagocytes interacted more with both neutrophils and T cells consistent with innate-adaptive crosstalk (Figure S13). Myeloid dysregulation has emerged as a major theme of severe or progressive COVID-19 and previous work reports both co-localisation of and crosstalk between cytotoxic CD8+ T cells with mononuclear phagocytes and neutrophils in areas of severe tissue damage.^33^ Other marked interactions included neutrophils (interferon signalling) and mononuclear phagocytes with inflammatory markers (IL1R^HI^, IL6R^HI^ and HLA-DR^HI^) (Figure S12). Compared to PRESneg tissue, PRESpos tissue showed increased interactions between or memory CD8+ T cells and a repair subset of macrophages (CD206^HI^) (Figure S12 and S13). M2 macrophages are known for their roles in tissue repair and reduce inflammation via suppression of effector T cells,^34^ indicating this process may start early, prior to overt tissue damage. Finally, B cells seemed to interact more with vascular endothelial cells in PRESpos compared to PRESneg, which may indicate early diapedesis and recruitment of antibody producing cells (Figure S12).

A more consistent interactome existed when comparing the DAD tissue phenotypes, however several notable changes were observed including 1) B cells/plasma cells increasing their interactions with mononuclear phagocytes and 2) T cells increasing their interactions with NK cells (Fig. 5, Figure S13).

## Discussion

In this study, we present a comprehensive assessment of the immune cell signature and structural cell composition in lung tissue from fatal COVID-19. Our data places particular emphasis on spatial and temporal differences in the heterogeneous patterns of tissue injury. We show that the pathological evolution of DAD, the archetypal lung pattern in COVID-19, is characterised by sustained increases in mononuclear phagocytes and lymphocytes without a shift in overall neutrophil counts but with shifts in functional neutrophil subsets. Within the structural compartments, there is a loss of AECs and endothelial cells. We confirm that significant airspace obliteration accompanies DAD progression and show this is a significant confounder in measuring relative and absolute cellularity in diseased lung tissue. Finally, critical immune and structural cell interactions occur prior to overt tissue injury.

The major immune cell shifts detected in the COVID-19 lung have been significant macrophage infiltration, expansion of T and B lymphocytes and mesenchymal and fibroblastic proliferation.^15^^,^^18^ However, interpretation of high resolution molecular pathology studies on COVID-19 PMLT has, to date, been limited by lack of discrimination between histologically different regions of interest^3^ in highly heterogeneous tissue.^3^^,^^8^^,^^9^^,^^35^ Rather, COVID-19 PMLT has hitherto been classified as ‘early’ or ‘late’ disease by chronological duration of illness,^15^ by comparison of viral negative and viral positive areas,^36^^,^^37^ by provision of an inflammation severity score,^2^ severity of tissue injury^33^ or simply being compared generally/collectively with non-infected control tissue.^19^ Our approach is instead based on the temporal phases of COVID-19 DAD evolution rooted in standardised pathological terminology first established by Katzenstein et al. in the 1970's.^38^ Erjefalt et al. (2022), using a multiplex immunohistochemistry platform also used the approach of discriminating between exudative, intermediate and organising DAD.^18^ Their results are consistent with our findings showing macrophages, B cells and both CD4+ and CD8+ T lymphocytes gradually increasing as DAD progresses.

Our findings confirm that mononuclear phagocyte infiltration is a major hallmark of COVID-19 lung tissue, proven across multiple modalities.^15^^,^^18^ Alongside a depletion of lung resident alveolar macrophages, there is a concurrent accumulation of pro-inflammatory mononuclear phagocytes which are peripherally recruited given correlation with cells in corresponding peripheral blood samples.^39^ Macrophage populations expressing pro-fibrotic genes similar to those found in idiopathic pulmonary fibrosis also accumulate.^40^ We observed progressive infiltration of both an inflammatory subset of monocyte/macrophages (IL1R^HI^, IL6R^HI^ and HLA-DR^HI^) as well as a CD206^HI^ monocyte/macrophage subset most likely to represent tissue repair macrophages with M2 polarisation^41^ as DAD progresses. The inflammatory group increased their interactions with interferon-responsive neutrophils early in disease progression, prior to overt tissue damage. This is interesting as prior imaging mass cytometry work noted a neutrophil pocket in an area with strong viral staining. Whilst this was in an area of severe tissue damage which were characterised partly by CD68+ macrophage infiltration, we may be detecting an early response signal.^33^ It is reasonable to hypothesise that select neutrophil populations respond early to viral presence and interact with mononuclear phagocytes to prime or augment their response thus establishing areas of severe tissue damage. The repair group of mononuclear phagocytes also interact with T lymphocytes early in disease progression, prior to overt tissue damage. This may implicate early innate-adaptive crosstalk in the establishment of the repair process. These early interactions may be critical in establishing dysregulated inflammatory responses and the over-zealous, deleterious repair processes which seem to be driven by macrophages. Proactive targeted modification of such interactions at disease detection may have some clinical application, if not for COVID-19 then for other viral illnesses which can lead to ARDS. Clinical implications likely transcend the lungs given mononuclear phagocyte infiltration in COVID-19 has been observed in other tissues ranging from the heart^42^ where they may be directly recruited by SARS-CoV-2-infected cardiomyocytes^43^ and even to perivascular niches in brain tissue.^44^

Similarly, our data shows that a second immune signature of COVID-19 DAD progression is a progressive infiltration of lymphocytes.^18^ This rise was accounted for by naïve and memory CD4+ T cells, memory CD8+ T cells, B cells and plasma cells. T cells, similar to monocytes/macrophages are thought to have dual roles in COVID-19, from a protective response in mild to moderate disease to a dysregulated one in severe cases.^45^ Lung resident and infiltrating B cells and plasma cells have received significantly less attention than T cells, although SARS-CoV-2-specific B cells have certainly been found in lung and lung-associated lymph nodal tissue.^46^ Increased B lymphocytes in COVID-19 BALF correlate with evidence of severe disease.^47^ Early recruitment and diapedesis of B lymphocytes/plasma cells is suggested from our results given their increased interaction with vascular endothelial cells in COVID positive compared to COVID negative tissue with preserved lung architecture. As DAD progresses however, there is continued plasma cell infiltration and increased B cell/plasma cell interactions with mononuclear phagocytes which may indicate a role in disease progression.

Lung tissue is inherently collapsible and subject to both anatomic variation in inflation as well as variance in tissue preparation for histological sectioning. Whilst attempts at standardisation by post-mortem lung formalin insufflation have previously been described,^48^ their routine use is impractical and unlikely to standardise inflation unless volume and pressure is personalised to the height and sex of the donor. In our study, all autopsies were performed under strict biohazard precautions meaning removal of the whole lungs and insufflation was also contraindicated. Variability in alveolar filling is notable across disease processes such as COVID-19 where alveolar type II cell destruction results in reduced surfactant production and reduced surface tension; airspace occlusion by oedema/haemorrhage/fibrin balls/neutrophil extracellular traps; and increased connective tissue production and contraction^49^ can all contribute. Underpinning our data is the ability to quantify absolute and relative cellularity in the PMLT. However, the conventional measure of cellularity, cells/mm^2^ of section area, used is confounded by variations in the area of sections occupied by airspace both in diseased and healthy lung. We showed that COVID-19 DAD progression is characterised by significant airspace obliteration, as reported in prior literature.^49^ Using the conventional metric of cells/mm^2^, alveolar epithelium and vascular endothelium increased throughout DAD. An increase in alveolar cells was unexpected given SARS-CoV-2 infects them^32^ and induces injury^38^ and apoptosis.^50^ However, application of an airspace correction factor showed a more plausible *decline* in alveolar epithelium as DAD progresses. A similar effect was also true of vascular endothelial cells. Application of an airspace correction factor showed a *decline* in vascular endothelium as DAD progresses. Whereas, the existing literature seems conflicted with observations of endothelialitis and endothelial apoptosis in COVID-19 PMLT^51^ but also increased vascular density, enhanced angiogenic gene expression and neoangiogenesis by intussusceptive angiogenesis.^52^^,^^53^ However, we are not the first to report a loss of endothelial cells specifically within areas of DAD^18^ and certainly disruption of the barrier function contributes to the formation of pulmonary oedema through outward movement of fluid and macromolecules.^54^ This discrepancy may be explained by mosaicism in vascular remodelling or damaged endothelial cells being undercounted by imaging mass cytometry. Regardless, this work confirmed that using cells/mm^2^ is subject to an artefact from progressively obliterated airspaces and increased cellular tissue in the region of interest. To our knowledge, correcting for airspace is rarely used despite providing valuable information and should be considered for analysis of pliable tissue. We suspect it is most appropriately used in disease processes where airspace variability is secondary to primary airspace obliteration such as with progressive DAD rather than bronchopneumonia in which neutrophils invade the alveolar space. It may also be used to correct inflation/deflation artefact where insufflation is not or cannot be used. Another limitation of airspace correction lies is that non-cellular space is not exclusively air and includes other non-cellular material such as oedema.

This study had several additional limitations. Clinical data available varied due to different collection across the three contributing biobanks; additionally, a subset died in the community with limited information prior to death; time to post-mortem varied and a level of tissue degradation might have occurred; though our control tissue appeared histologically normal, it was obtained from declined donor lungs and therefore by definition deemed unsuitable for transplant; due to the scarcity of organ donor lungs during the study period owing to the pandemic stage only two control donors were utilised however this was mitigated by using 15 ROIs from each donor; our antibody panel was not designed to target markers of fibrosis; there was no capability to confirm the specific SARS-CoV-2 variant each person was affected by therefore generalisability to current or future variants may be limited; finally, there would be a significant amount of confounding in this study which could not be controlled for example in hospital-based care where various treatments may have affected the immune response.

In summary, we have presented a comprehensive assessment of the cellular signature of COVID-19 DAD progression using an airspace correction method to normalise cell counts and account for a progressive march towards airspace obliteration. Sustained increases in MnPs and lymphocytes and a loss of AECs and endothelial cells are the hallmark of COVID-19 DAD progression and although neutrophils were overall stable, there is a shift in several functional subsets. Finally, we performed a neighbourhood analysis focussed on the distinct temporal phases of DAD progression and identified that critical immune cell interactions occur early in the disease process, prior to overt tissue damage.

## Contributors

**Luke Milross MD**: conceptualization, formal analysis, investigation, data curation, writing—original draft, visualisation; **Bethany Hunter**: methodology, validation, formal analysis, investigation, data curation, writing—original draft, visualisation; **David McDonald PhD**: formal ani.ealysis, investigation, data curation, writing—review & editing; **George Merces PhD**: methodology, software, formal analysis, data curation, writing—review & editing; **Amanda Thomson**: validation, writing—review & editing; **Catharien M.U. Hilkens**: data curation, writing—review & editing; **John Wills PhD**: software, formal analysis, writing—review & editing; **Paul Rees PhD**: software, formal analysis, writing—review & editing; **Kasim Jiwa**: resources, writing—review & editing; **Nigel Cooper**: resources, writing—review & editing; **Joaquim Majo**: cenceptualization, methodology, formal analysis, data curation, writing—review & editing; **Helen Ashwin**: data curation, writing—review & editing; **Christopher JA Duncan DPhil**: resources, validation, writing—review & editing; **Paul M Kaye PhD**: conceptualization, methodology, writing—review & editing; **Omer Bayraktar PhD**: conceptualization, methodology, writing—review & editing; **Andrew Filby PhD**: conceptualization, methodology, software, validation, formal analysis, investigation, resources, data curation, writing—original draft, writing—review & editing, visualisation, supervision, project administration; and **Andrew J. Fisher PhD FRCP**: conceptualization, methodology, formal analysis, resources, writing—original draft, writing—review & editing, supervision, project administration. Luke Milross, Bethany Hunter, David McDonald, George Merces, Andrew Filby and Andrew J. Fisher were involved in verification of the underlying data. All authors read and approved the final version of the manuscript.

## Data sharing statement

The datasets generated for this study are available on reasonable request through contact to the following address: andrew.filby@newcastle.ac.uk.

## Declaration of interests

L Milross was supported by a General Sir John Monash Scholarship awarded by the General Sir John Monash Foundation and a Vice-Chancellor's Global Scholarship from Newcastle University in support of a Master of Research in Immunobiology at Newcastle University. A Thomson was supported by funding from the JGW Patterson Foundation. C. J. A. Duncan was supported by a Wellcome Clinical Research Career Development Fellowship (211153/Z/18/Z).
