# Supplementary material for: Distinct lung cell signatures define the temporal evolution of diffuse alveolar damage in fatal COVID-19
Source: eBioMedicine. 2023 Dec 23;99:104945. doi: 10.1016/j.ebiom.2023.104945 (PMC10788437; doi:10.1016/j.ebiom.2023.104945)

Suppl. Figure 1

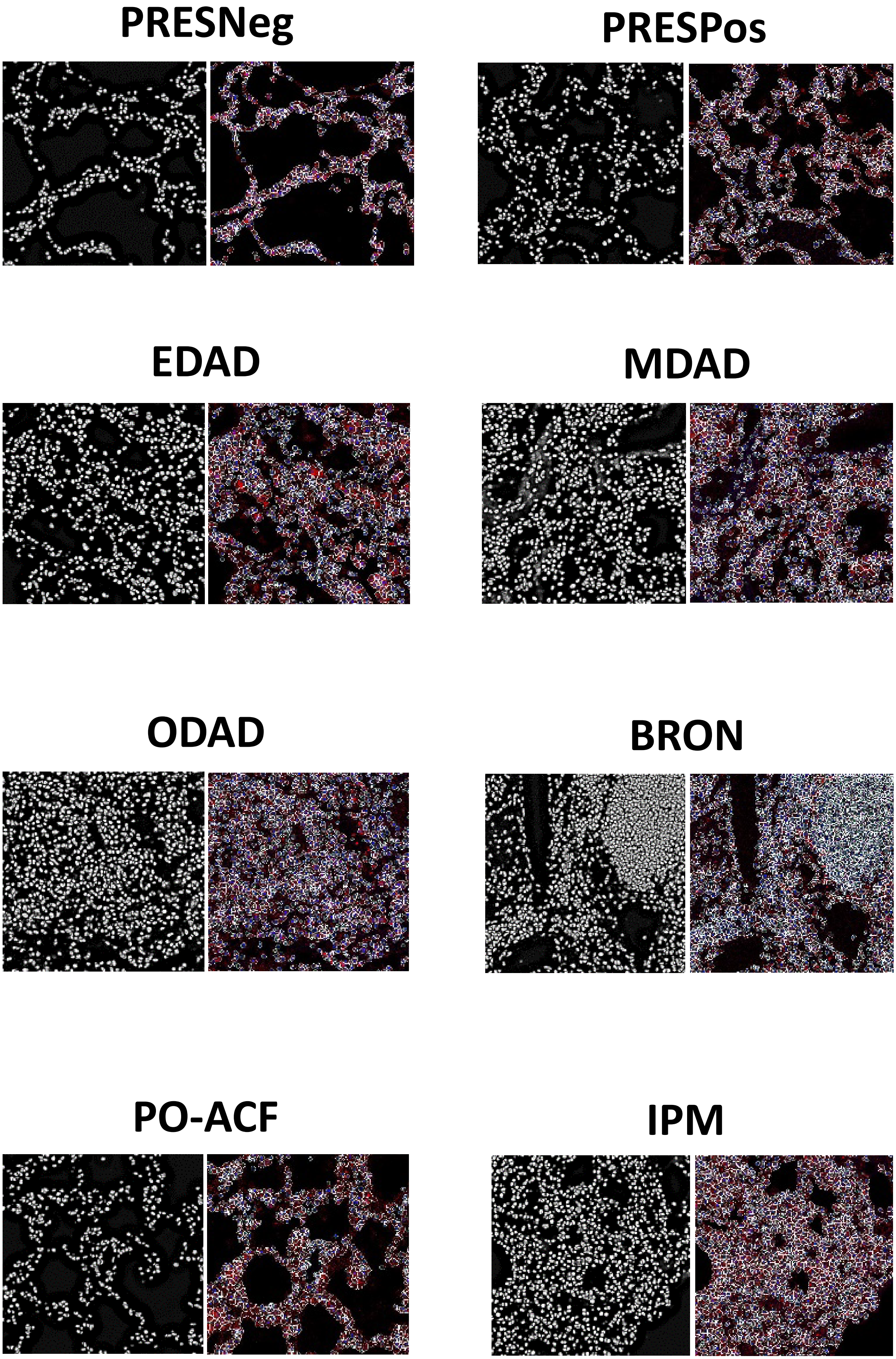

Suppl. Figure 2

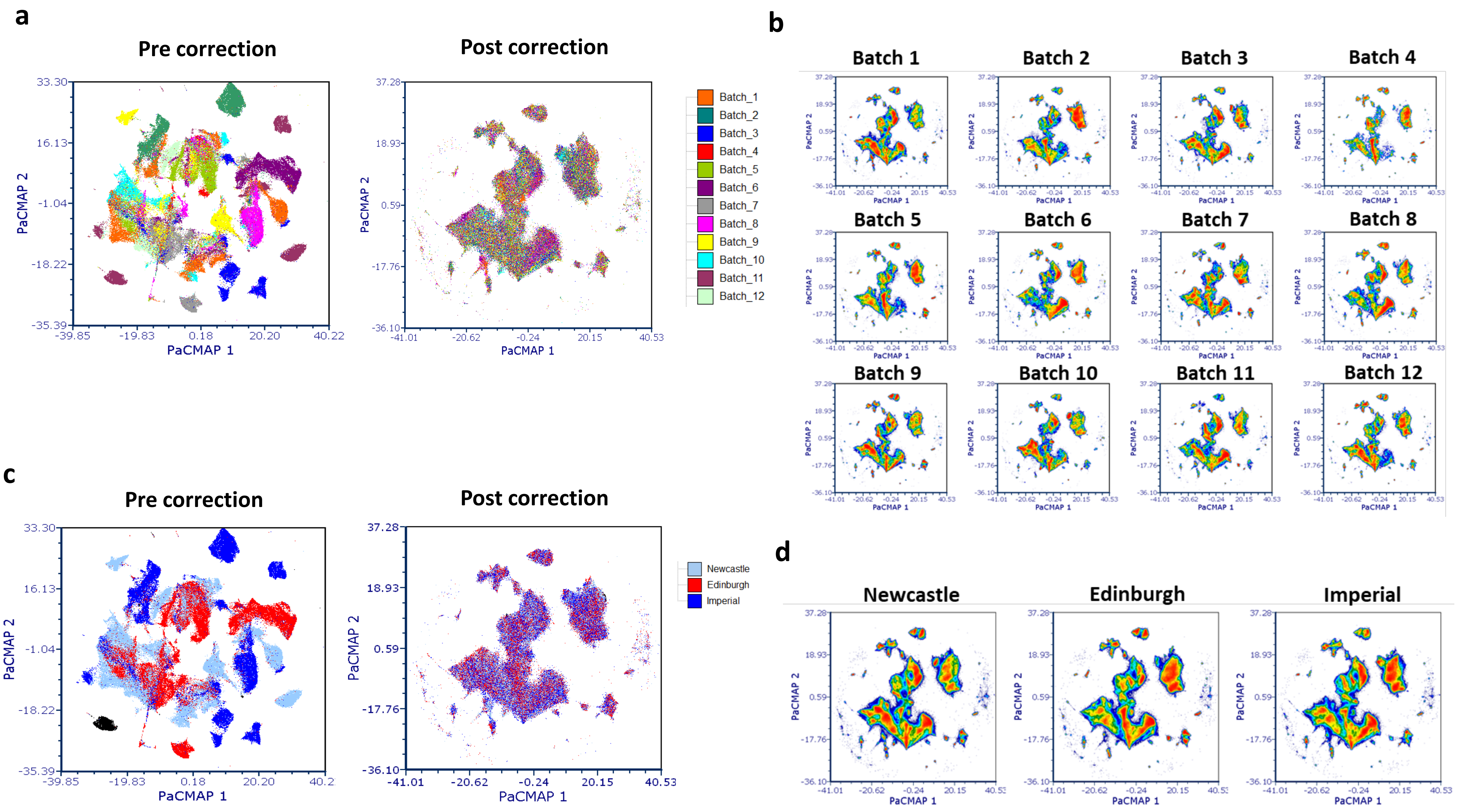

Suppl. Figure 3

a

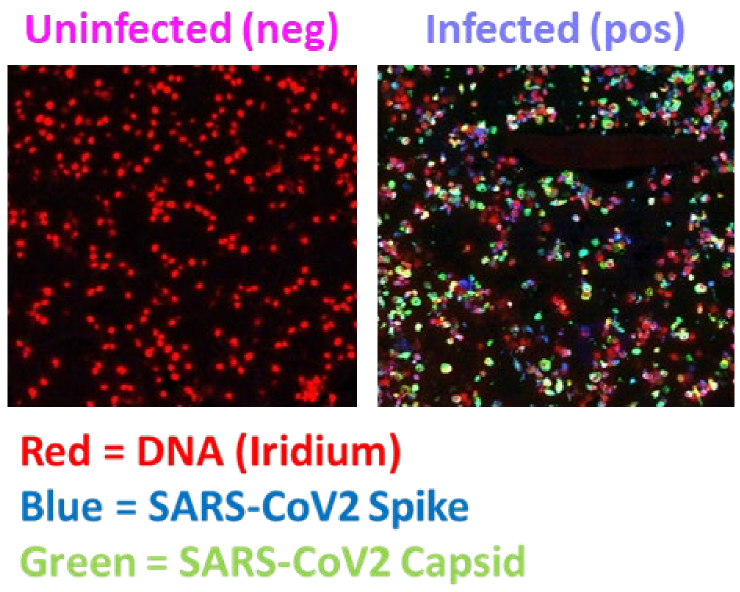

b

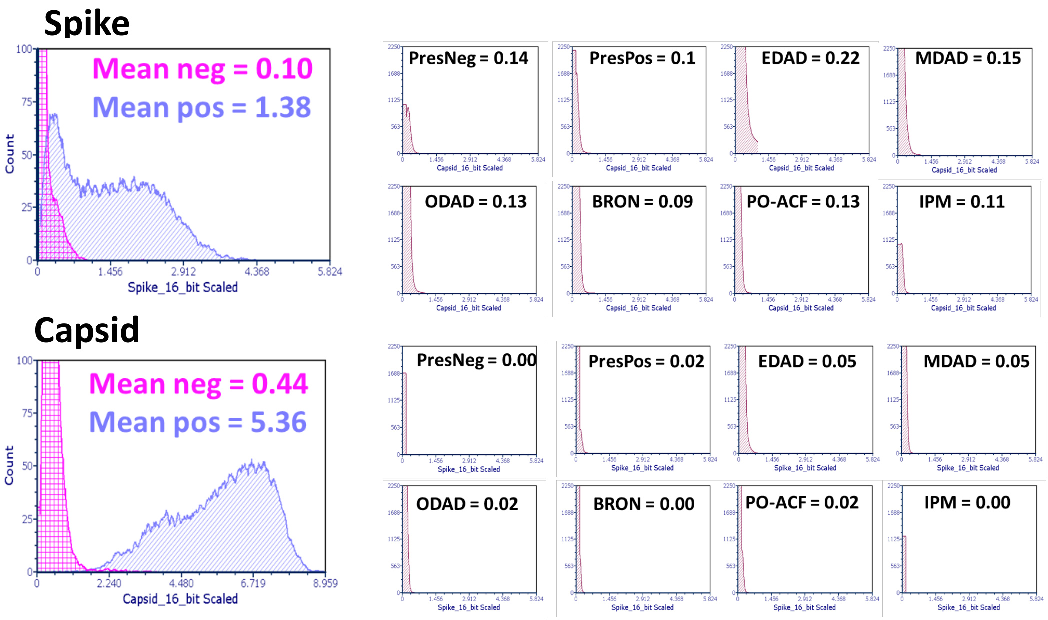

Suppl. Figure 4

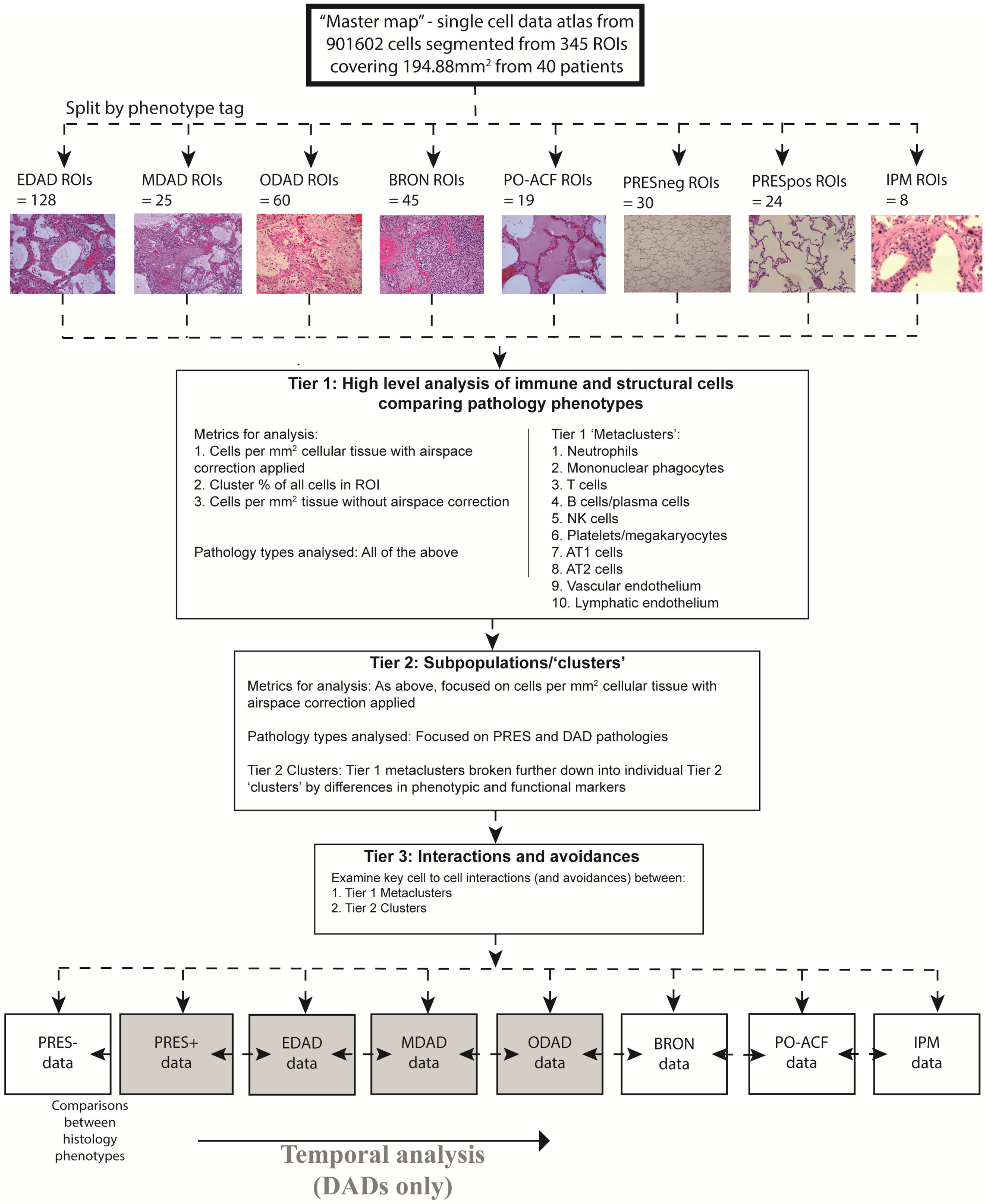

Suppl. Figure 5

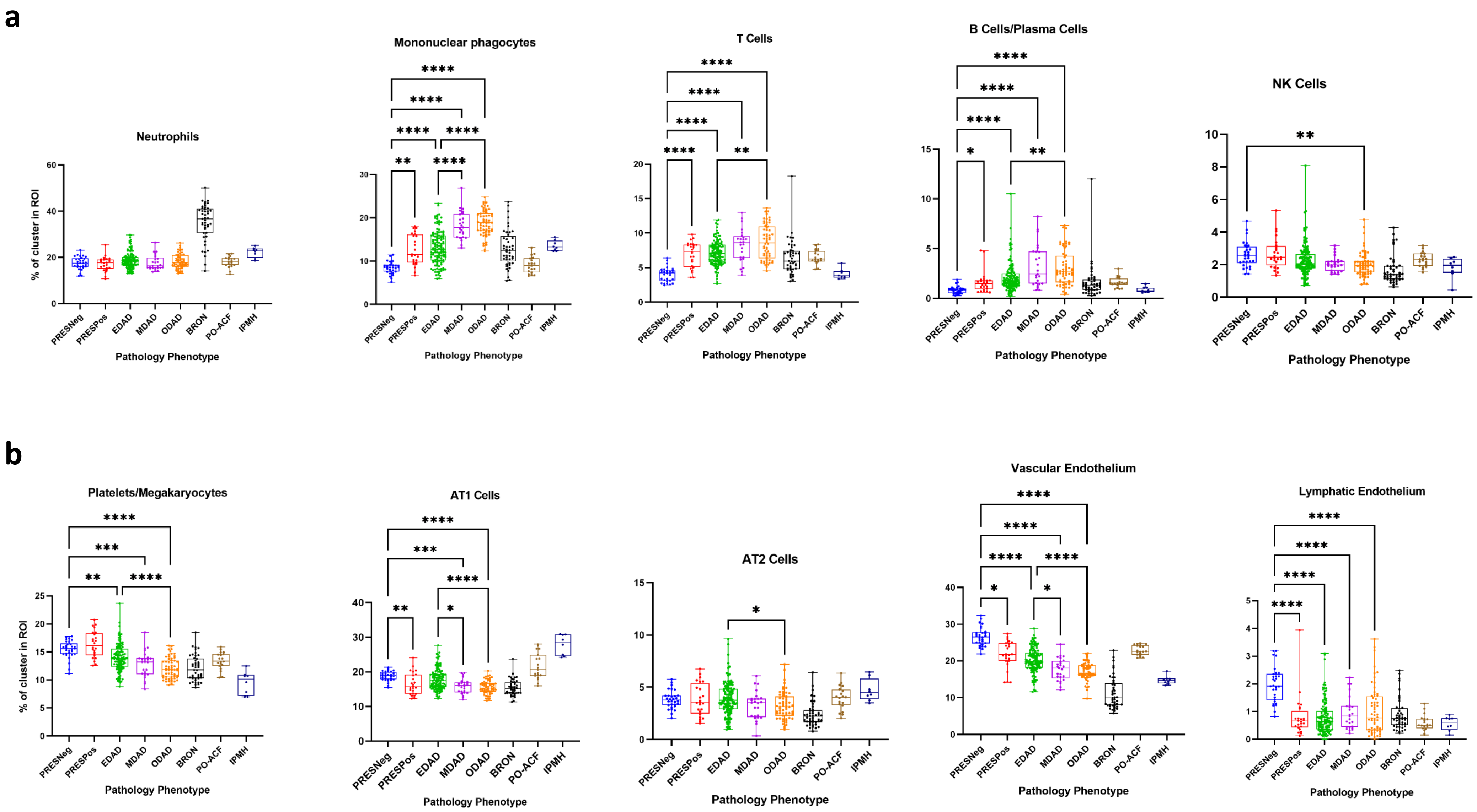

Suppl. Figure 6

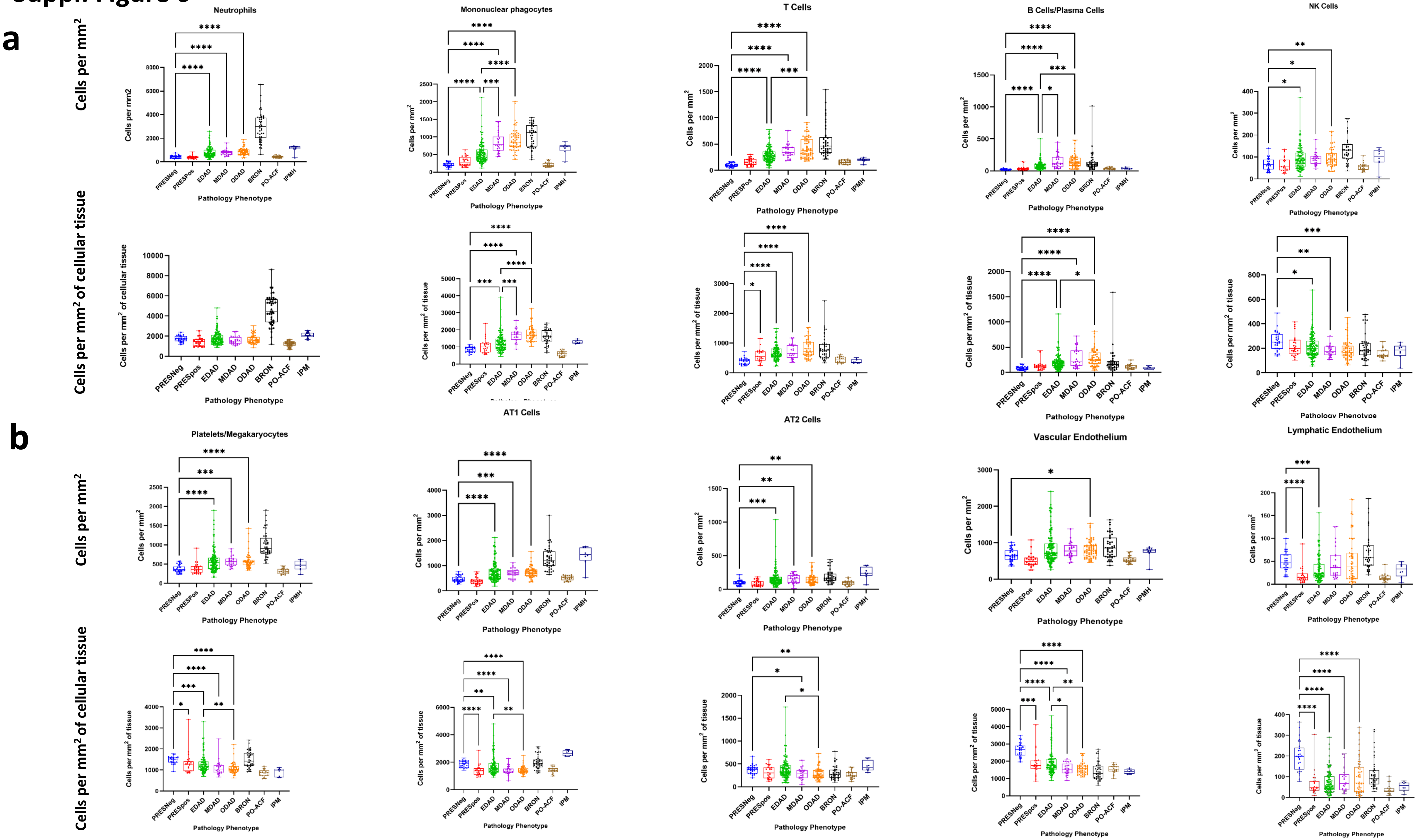

Suppl. Figure 7

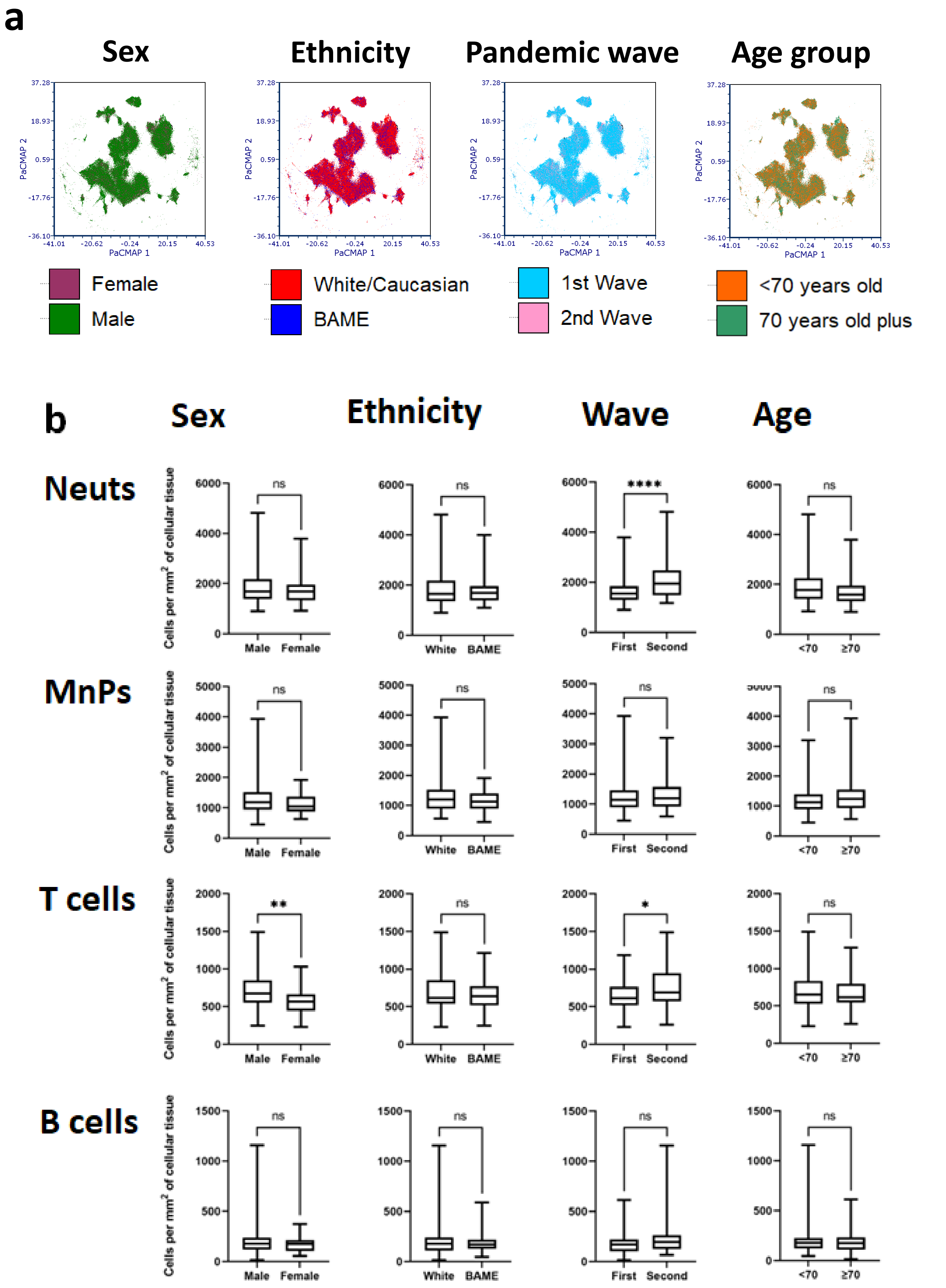

Suppl. Figure 8

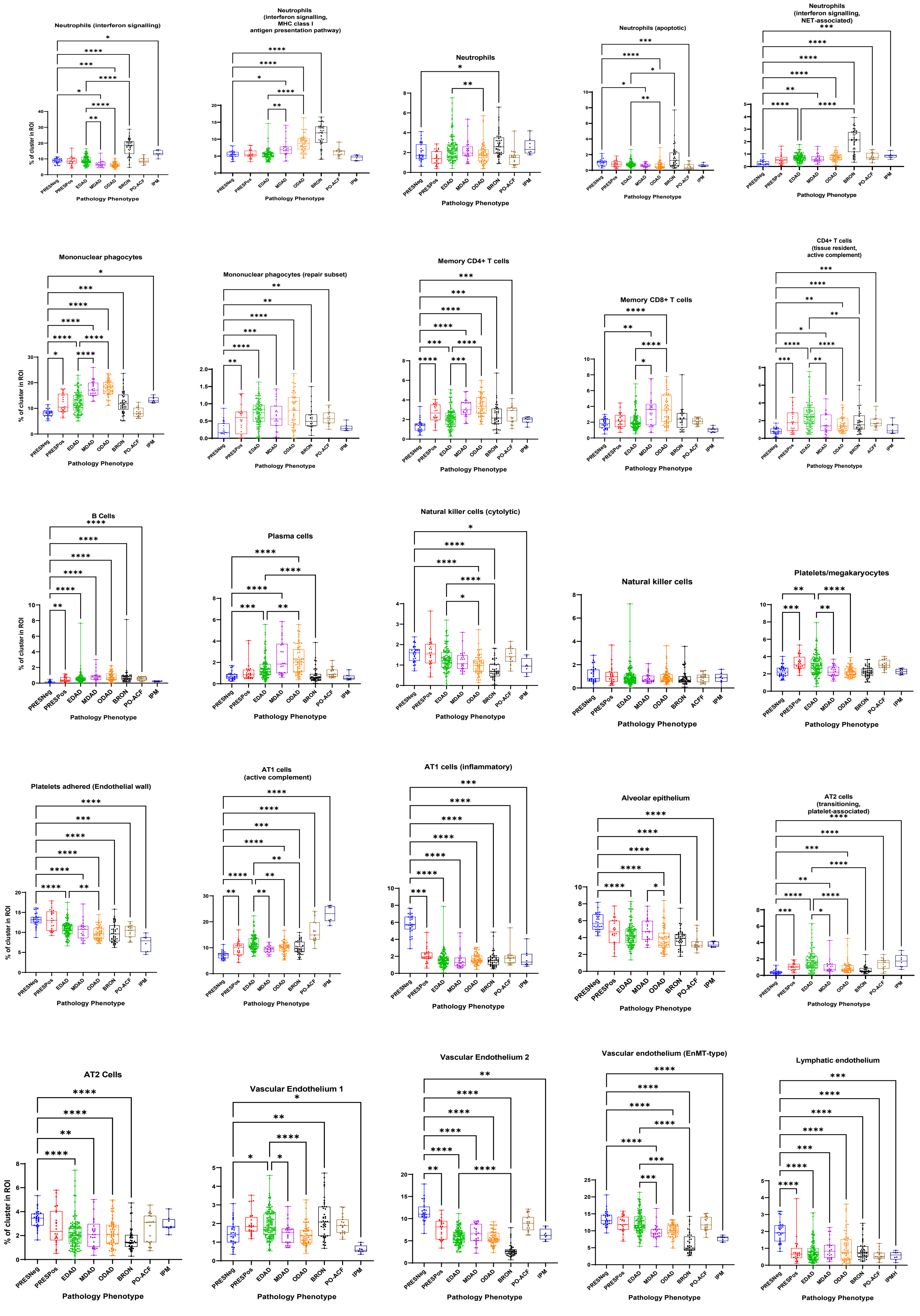

Suppl. Figure 9

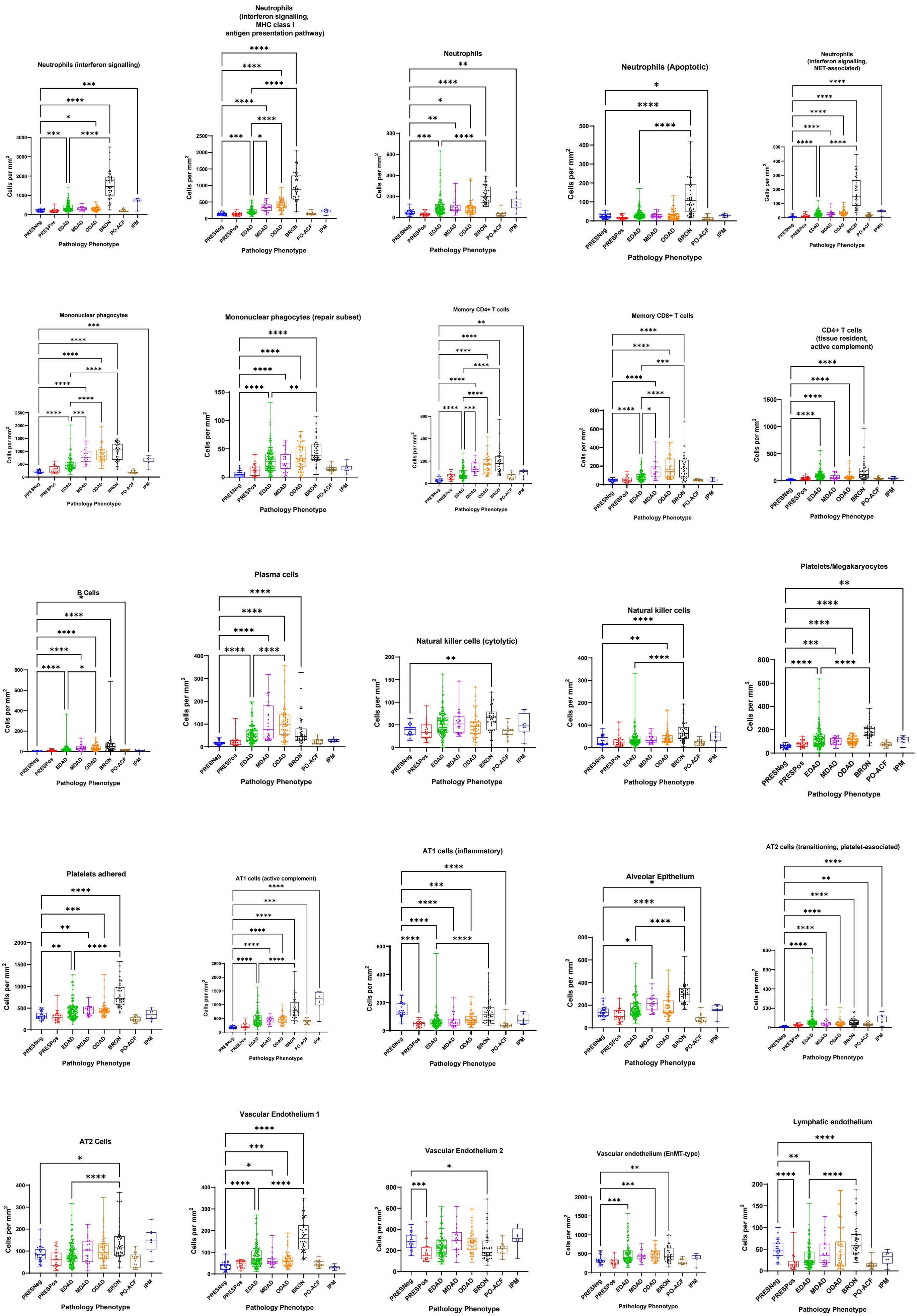

Suppl. Figure 10

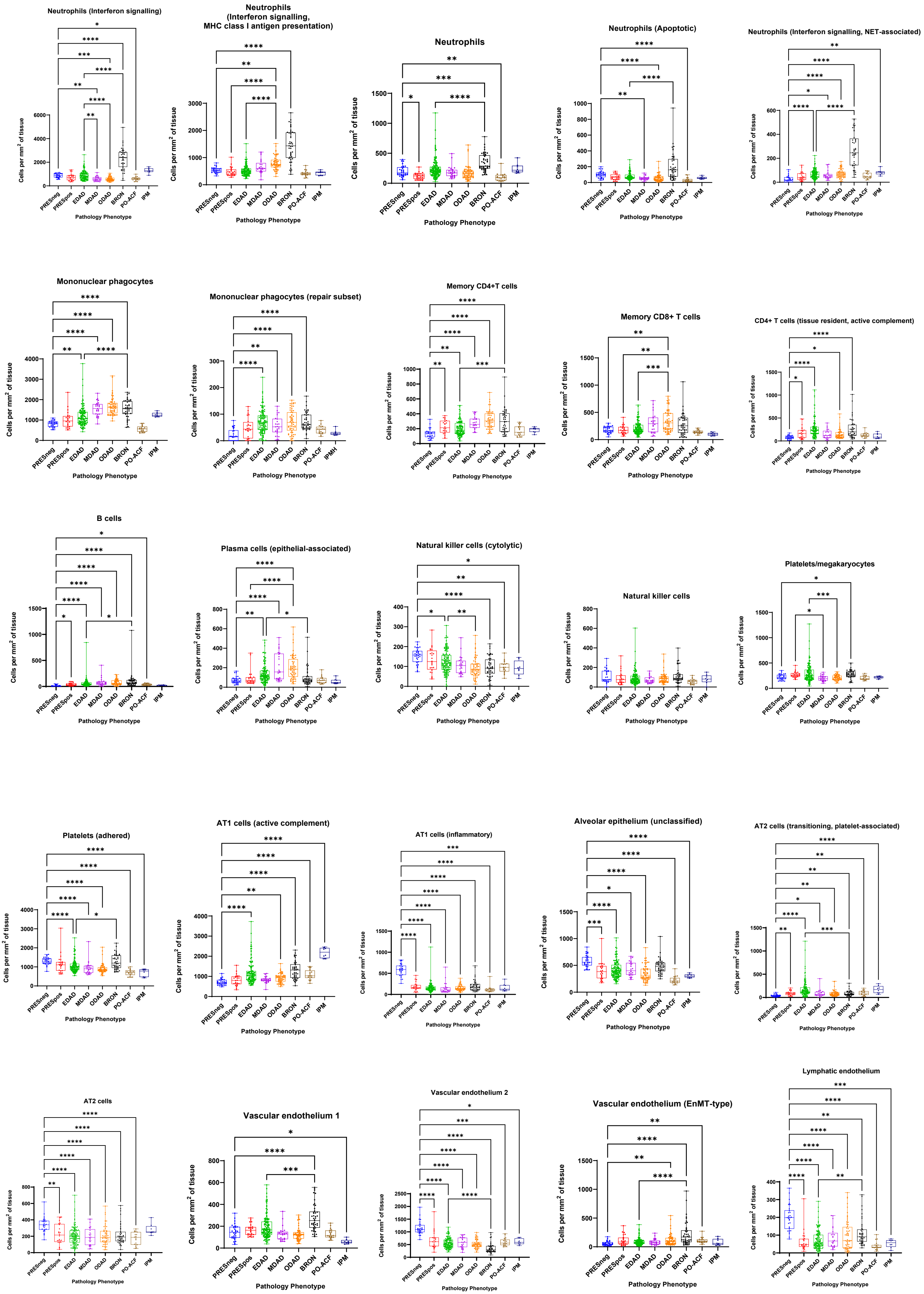

Suppl. Figure 11

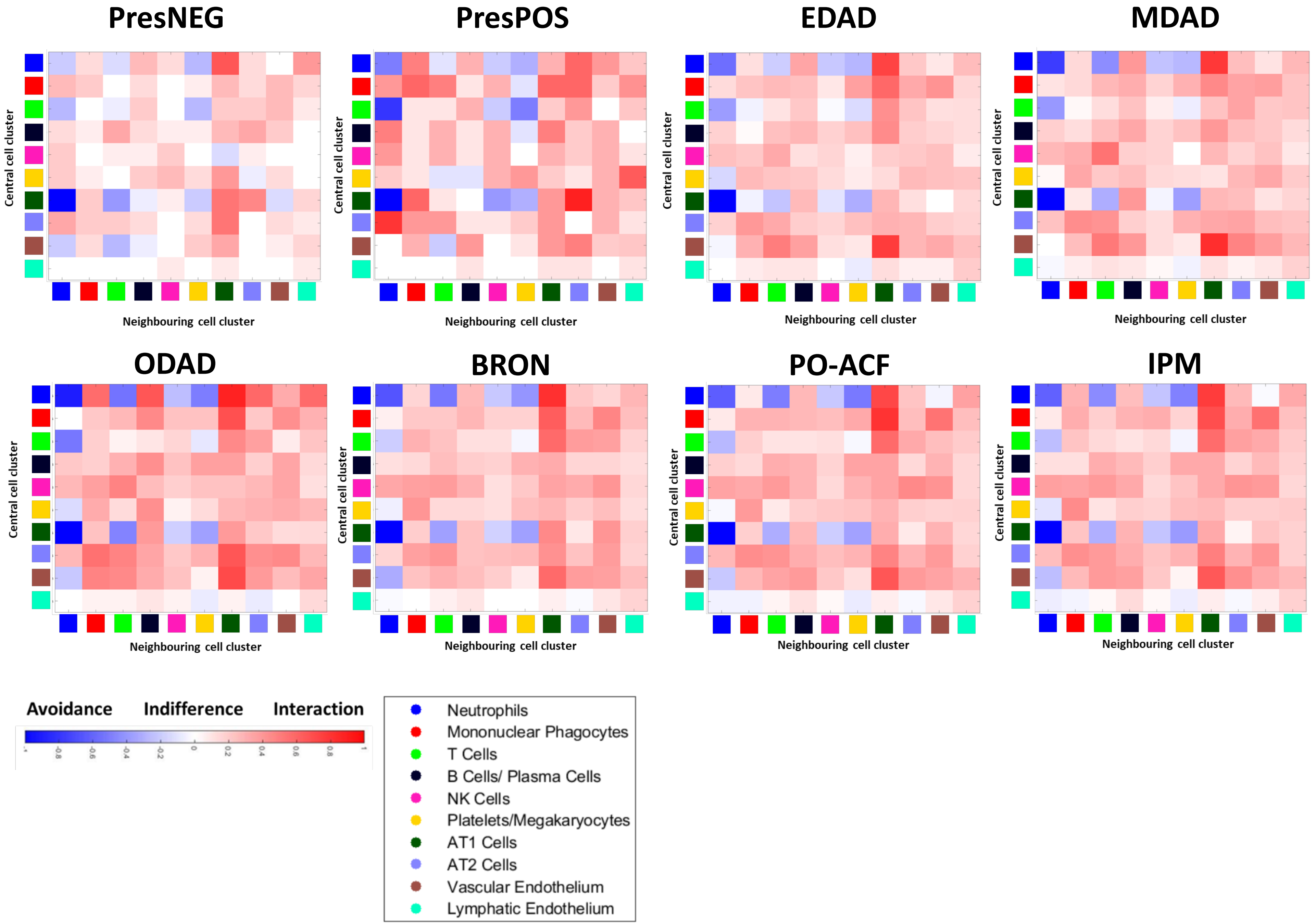

Suppl. Figure 12

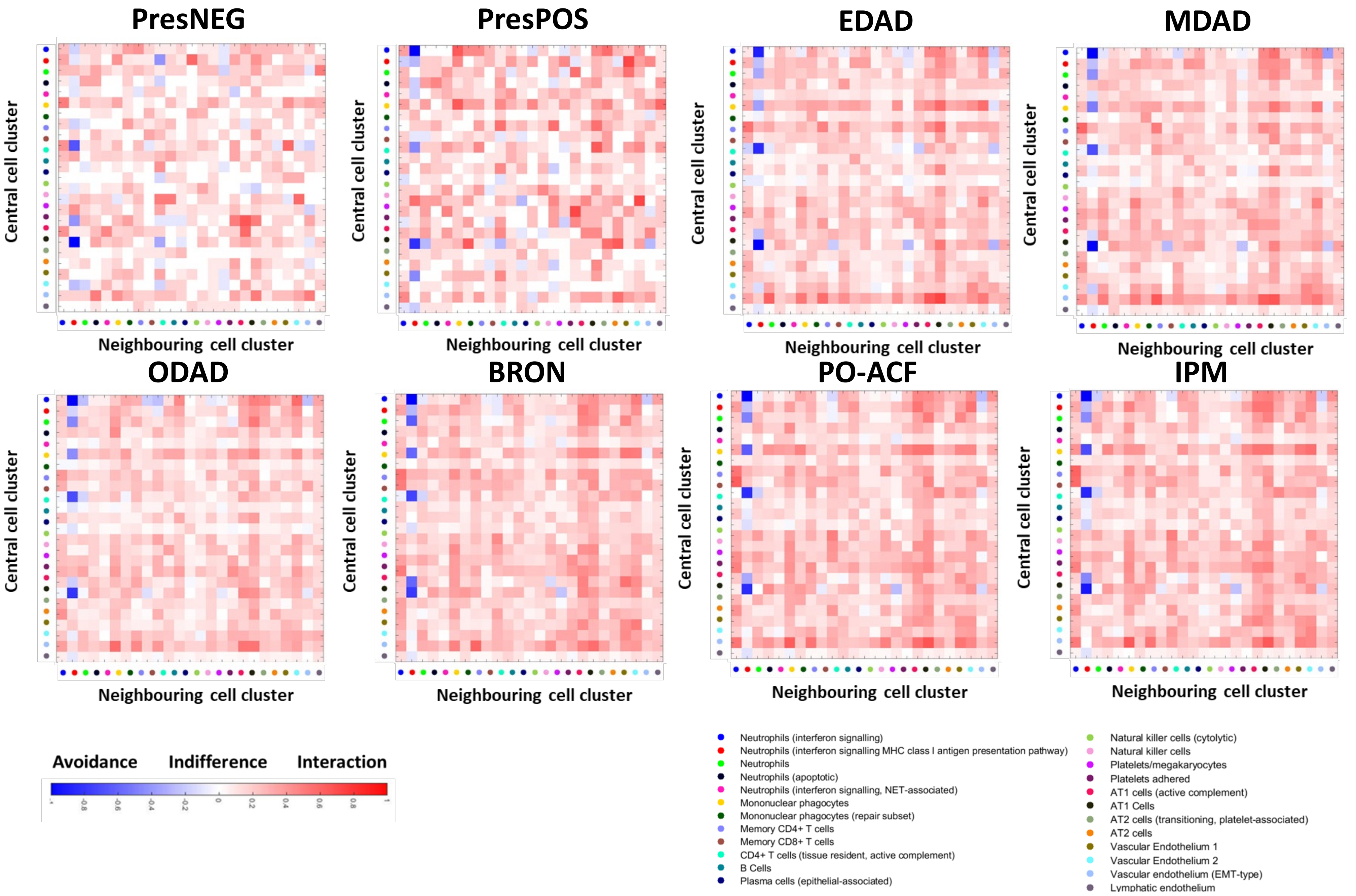

Suppl. Figure 13

a

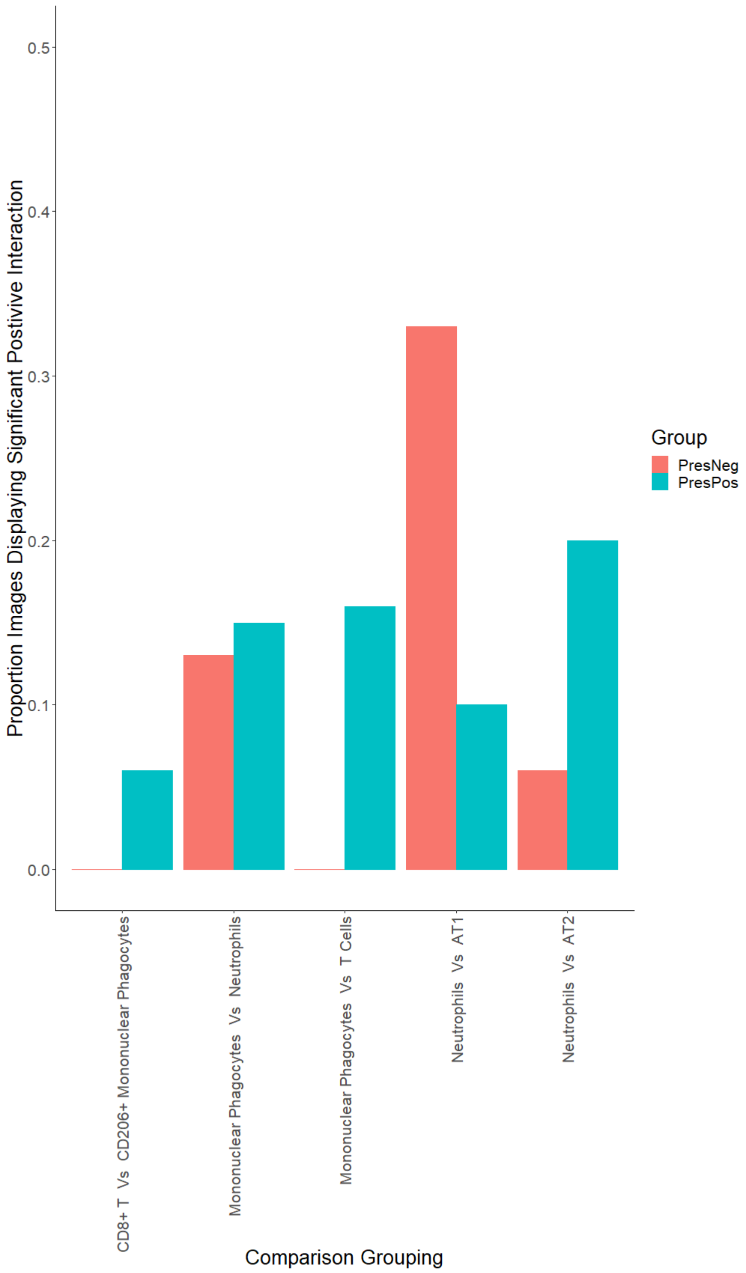

b

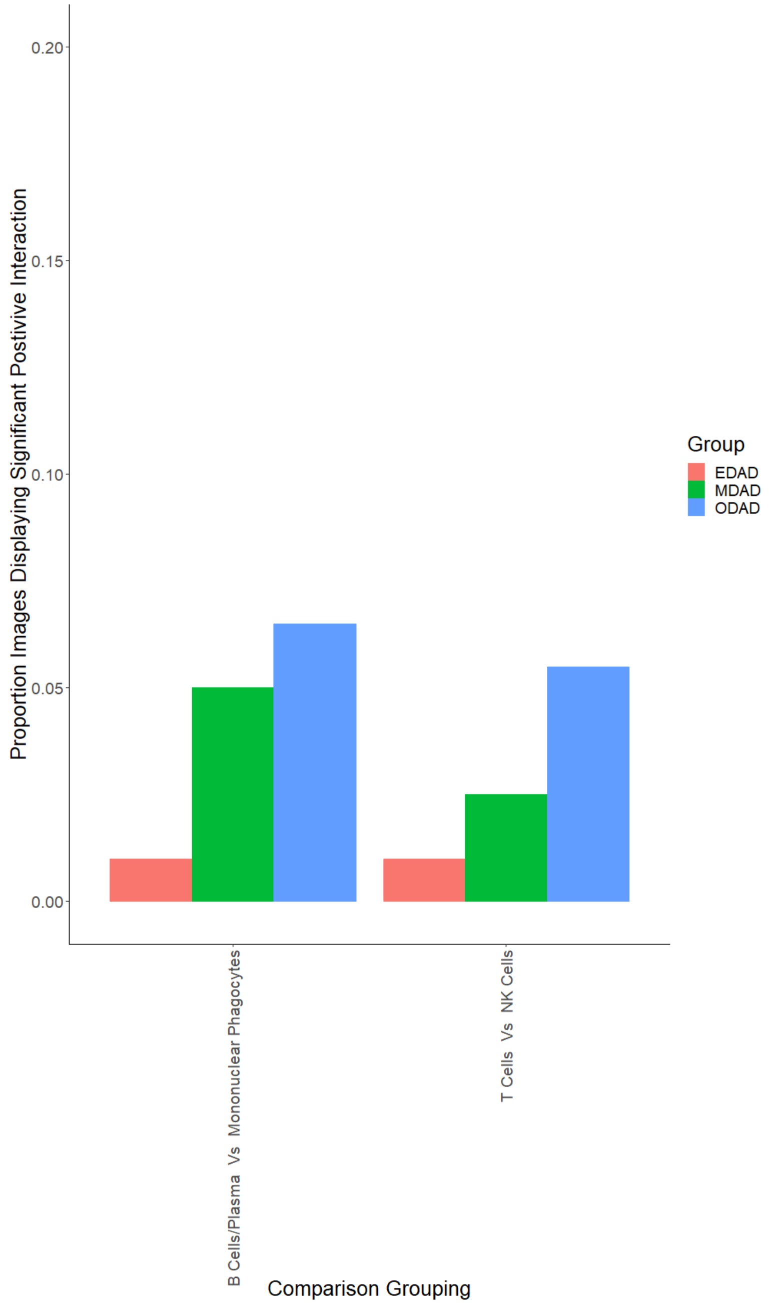

Supplement: Figures S1–S13 [file mmc1.pdf]
