# Supplementary material for: Distinct lung cell signatures define the temporal evolution of diffuse alveolar damage in fatal COVID-19
Source: eBioMedicine. 2023 Dec 23;99:104945. doi: 10.1016/j.ebiom.2023.104945 (PMC10788437; doi:10.1016/j.ebiom.2023.104945)
Supplement: Tables S1–S4 [file mmc2.docx]

**Supp Table 1.** *Disease characteristics of the cohort*

| **Disease Course** | |
| --- | --- |
| **Duration of Illness (n=40)** | |
| Median (days) | 12 |
| Interquartile range (days) | 8.75-22.25 |
| ≤15 days | 21 (52.5%) |
| >15 days | 14 (35%) |
| Unknown | 5 (12.5%) |
| **Symptoms (n= 40)** | |
| Respiratory | 30 (75%) |
| Enteric | 7 (17.5%) |
| Fever | 22 (55%) |
| Systemic | 23 (57.5%) |
| Unknown | 3 (7.5%) |
| **Exposure to medications** | |
| Corticosteroids (n=38) | 10 (26.3%) |
| Antibiotics (n=39) | 25 (64.1%) |
| Anticoagulation (n=37) | 20 (54.1%) |

**Supp Table 2.** *Regions of interest selected for each histological pattern*

| **Predominant Histology Phenotypes (n=40)** | |
| --- | --- |
| EDAD | 128 |
| MDAD | 25 |
| ODAD | 60 |
| BRON | 45 |
| PO-ACF | 19 (from 3 cases) |
| IPM | 8 (from 1 case) |
| PRESneg | 30 (from 2 cases) |
| PRESpos | 24 |

**Supp Table 3.** *Histological Criteria utilised for pathology phenotype identification and ROI selection*

| **Pathology Code** | **Hallmark Characteristic** | **Supportive Features** |
| --- | --- | --- |
| EDAD | Presence of hyaline membranes (HM)  Absence of fibroblastic organisation (FO) | Mild interstitial inflammation  Type II pneumocyte hyperplasia  Alveolar haemorrhage |
| ODAD | Presence of FO  Absence of HM | Fibroblast aggregates  Scattered inflammatory cells  Collagen deposition  Squamous metaplasia |
| MDAD | Presence of HM and FO in any degree |  |
| BRON | Neutrophilic exudate | +/- tissue damage  Alveolar haemorrhage |
| PO-ACF | Alveolar / interstitial oedema  Absence of tissue injury | Pulmonary vascular congestion |
| IPM | Identification of fungal hyphae  Tissue inflammation / necrosis | Angioinvasion  Positive culture |

**Supp Table 4.** *Antibodies, clones and conjugates*

| **Target** | **Clone** | **Vendor** | **Catalogue #** | **RRID Tag** | **Metal** | **Working Concentration**  **(ug/mL)** | **Major target/cell type/population** | **Main spatial location** |
| --- | --- | --- | --- | --- | --- | --- | --- | --- |
| SARS-CoV-2  Spike | E7M5X | Cell Signalling Technology | 42172S | AB_2941940 | 113In | 5 | SARS-CoV-2 virus, Spike protein | Various |
| CD45RO | UCHL1 | Thermofisher | 14-0457-82 | AB_467270 | 115In | 4 | Memory T cells (+ macrophages) | Immune cell marker, surface |
| CD45RA | HI100 | Thermofisher | 14-0458-82 | AB_467272 | 141Pr | 4 | Naïve T cells | Immune cell marker, surface |
| CD68 | KP1 | Biolegend | 916104 | AB_2616797 | 142Nd | 5 | Tissue macrophages (+ monocytes and monocyte- derived dendritic cells) | Immune cell marker, surface |
| CD8a | C8/144B | Biolegend | 372902 | AB_2650657 | 143Nd | 6 | Cytotoxic T cells | Immune cell marker, surface |
| KI67 | Polyclonal | Novus | NB500-170 | AB_1660251 | 144Nd | 3 | Proliferating cells | Non-specific functional marker, nuclear |
| Collagen I | 3D5E8 | Protein Tech | 66761-1-Ig | AB_2882107 | 145Nd | 2.5 | Connective tissues including extracellular matrix | Structural, extracellular |
| CD138 | 4F3A8 | Protein Tech | 67155-1-Ig | AB_2882452 | 146Nd | 3.5 | Plasma cells or precursors to plasma cells (plasmablasts) | Immune cell marker, surface |
| CD163 | EDHu-1 | BioRad | MCA1853 | AB_2074540 | 147Sm | 10 | Monocytes/macrophages and monocyte-derived dendritic cells | Immune cell marker, surface |
| IL1-R | Polyclonal | Thermofisher | PA5-28834 | AB_2546310 | 148Nd | 3.5 | Multiple cell types | Cell surface |
| CD42b | SP219 | Abcam | ab240268 | AB_2814749 | 149Sm | 8 | Platelets, megakaryocytes | Platelet cell surface, cytosolic in megakaryocytes. |
| MPO | 4C11F6 | Protein Tech | 66177-1-Ig | AB_2881572 | 150Nd | 5 | Polymorphonuclear leucocytes/granulocytes (neutrophils, basophils, eosinophils), monocytic cells. | Immune cell marker, cytosolic (lysosome) |
| SAR-Cov-2  Capsid | E8R1L | Cell Signalling Technology | 33717S | AB_2941972 | 151Eu | 5 | SARS-CoV-2 virus, Capsid protein | Various |
| B7 | B7 (Kemp et al., 1992) | Gifted Paul  Morgan | NA | N/A | 152Sm | 20 | Antigen presenting cells | Cell surface, functional marker |
| CD56 | E7X9M | Cell Signalling Technology | 99746BF | AB_2868490 | 153Eu | 12 | Natural killer cells (+*alpha beta T cells, gamma delta T cells, dendritic cells, and monocytes*) | Immune cell marker, surface |
| Podoplanin | NZ-1.3 | Thermofisher | 14-9381-82 | AB_1603307 | 154Sm | 6 | Lymphatic endothelium | Structural, cell surface |
| CD69 | 15B5G2 | Novus | NBP2-25236 | N/A | 155Gd | 0.25 | Tissue-restricted T cells, NK cells, other immune cells, and platelets. | Immune cell marker, surface |
| EPCAM | Polyclonal | Abcam | ab71916 | AB_1603782 | 156Gd | 1 | Most epithelial cells (such as airway epithelial cells and  pneumocytes) | Cell surface, particularly basolateral  surface of epithelial cells |
| CD206 | 2A6A10 | Protein Tech | 60143-1-Ig | AB_2144924 | 157Gd | 0.5 | Macrophages and dendritic cells, associated with M2 macrophage polarisation | Immune cell marker, surface |
| CD79a | EP3618 | Abcam | ab239891 | AB_2260147 | 158Gd | 7.5 | B cells | Immune cell marker, surface |
| STING | D2P2F | Cell Signalling Technology | 13647 | AB_2732796 | 159Tb | 5 | Ubiquitous, IFN responding cells | Ubiquitous. Cell membrane and endoplasmic reticulum |
| TMPRSS | Polyclonal | Thermofisher | PA5-14265 | AB_2272011 | 160Gd | 10 | Expressed by multiple human cell types including but not limited to airway epithelial cells and pneumocytes.  Facilitator of SARS-CoV-2 viral entry. | Cell surface |
| AQP5 | Polyclonal | Thermofisher | PA5-14264 | AB_2272011 | 161Dy | 5 | Type I pneumocytes (and other cell types including multiple immune cells) | Structural - Cell surface marker for Type I pneumocytes, apical membrane distribution |
| CD1c | 2A7C11 | Novus | NBP2-61726 | N/A | 162Dy | 2.5 | Dendritic cells, monocytes and monocyte-derived dendritic cells (and *B cells)* | Immune cell marker, surface |
| IFITM3 | Polyclonal | Protein Tech | 11714-1-AP | AB_2295684 | 163Dy | 5 | Ubiquitous, IFN responding cells | Non-specific functional marker, cell  membrane and endolysosomal |
| ACE2 | 2F12A4 | Protein Tech | 66699-1-Ig | AB_2882052 | 164Dy | 5 | Expressed by multiple human cell types including but not limited to airway epithelial cells and pneumocytes.  Facilitator of SARS-CoV-2 viral entry. | Cell surface |
| CD57 | HNK-1 | Biolegend | 359602 | AB_2562403 | 165Ho | 10 | T cells, NK cells | Cell surface, functional marker |
| p16 | EPR1473 | Abcam | ab186932 | AB_2895712 | 166Er | 10 | Ubiquitous, marker of cell senescence | Cytosolic/Nuclear |
| IL6-R | Polyclonal | Thermofisher | PA5-100836 | AB_2850337 | 167Er | 10 | Multiple cell types | Cell surface |
| Cleaved Caspase-3 | Asp175 | Cell Signalling Technology | 9579S | AB_10897512 | 168Er | 5 | Apoptotic cells | Non-specific functional marker, cytoplasmic |
| CD61 | D7X3P | Cell Signalling Technology | 13166BF | AB_2798136 | 169Tm | 5 | Megakaryocytes, platelets | Cell surface |
| CD3 | Polyclonal | Fluidigm | 3170019D | AB_2811048 | 170Er | 7.5 | T cells | Immune cell marker, surface |
| ProSPC | Polyclonal | Novus | NBP1-60117 | AB_11029499 | 171Yb | 4 | Type II pneumocytes | Cytoplasmic |
| CD31 | EPR3094 | Abcam | ab207090 | AB_2889382 | 172Yb | 2 | Platelets, endothelial cells (and *leukocytes*) | Cell surface |
| C3-30 | C3-30 (Kemp et al., 1992) | Gifted Professor Claire Harris | N/A | N/A | 173Yb | 8 | Complement component C3 (activated complement) | Extracellular/cell membrane/ubiquitous |
| CD4 | EPR6855 | Abcam | ab181724 | AB_2864377 | 174Yb | 6 | T helper cells (*and NK T cells, innate lymphoid cells and macrophages*). | Immune cell marker, surface |
| HLA-DR | LN3 | Thermofisher | 14-9956-82 | AB_468639 | 175Lu | 5 | ‘Professional’ APCs including B cells, DCs, macrophages, monocytes | Immune cell marker, surface |
| CD169 | SP213 | Abcam | ab245735 | AB_2864563 | 176Yb | 2.5 | Macrophages (and dendritic cells) | Immune cell marker, surface |
| CD147 | E1S1V | Cell Signalling Technologies | 13287BF | AB_2798171 | 194Pt | 4 | Widely expressed in multiple human cell types including leucocytes, epithelial cells and endothelial cells.  Alternate facilitator of SARS-CoV-2 viral entry. | Cell surface |
| Beta-2- microglobulin | EPR21752- 214 | Abcam | ab237032 | N/A | 198Pt | 1.5 | All nucleated cells | Cell surface |
